# The role of epidemic spreading in seizure dynamics and epilepsy surgery

**DOI:** 10.1162/netn_a_00305

**Published:** 2023-06-30

**Authors:** Ana P. Millán, Elisabeth C. W. van Straaten, Cornelis J. Stam, Ida A. Nissen, Sander Idema, Johannes C. Baayen, Piet Van Mieghem, Arjan Hillebrand

**Affiliations:** Amsterdam UMC, Vrije Universiteit Amsterdam, Department of Clinical Neurophysiology and MEG Center, Amsterdam, The Netherlands; Amsterdam UMC, Vrije Universiteit Amsterdam, Department of Neurosurgery, Amsterdam, The Netherlands; Amsterdam Neuroscience, Brain Imaging, Amsterdam, The Netherlands; Amsterdam Neuroscience, Systems and Network Neurosciences, Amsterdam, The Netherlands; Amsterdam Neuroscience, Neurodegeneration, Amsterdam, The Netherlands; Amsterdam Neuroscience, Cancer Biology and Immonology, Amsterdam, The Netherlands; Amsterdam Neuroscience, Imaging and Biomarkers, Amsterdam, The Netherlands; Faculty of Electrical Engineering, Mathematics and Computer Science, Delft University of Technology, Delft, The Netherlands

**Keywords:** Epilepsy surgery, MEG brain networks, Seizure modeling, Epidemic spreading model, Personalized medicine

## Abstract

Epilepsy surgery is the treatment of choice for drug-resistant epilepsy patients, but only leads to seizure freedom for roughly two in three patients. To address this problem, we designed a patient-specific epilepsy surgery model combining large-scale magnetoencephalography (MEG) brain networks with an epidemic spreading model. This simple model was enough to reproduce the stereo-tactical electroencephalography (SEEG) seizure propagation patterns of all patients (*N* = 15), when considering the resection areas (RA) as the epidemic seed. Moreover, the goodness of fit of the model predicted surgical outcome. Once adapted for each patient, the model can generate alternative hypothesis of the seizure onset zone and test different resection strategies in silico. Overall, our findings indicate that spreading models based on patient-specific MEG connectivity can be used to predict surgical outcomes, with better fit results and greater reduction on seizure propagation linked to higher likelihood of seizure freedom after surgery. Finally, we introduced a population model that can be individualized by considering only the patient-specific MEG network, and showed that it not only conserves but improves the group classification. Thus, it may pave the way to generalize this framework to patients without SEEG recordings, reduce the risk of overfitting and improve the stability of the analyses.

## Author Summary

In this study we showed that epidemic spreading models that utilise patient-specific data of brain connectivity capture the main aspects of seizure propagation as clinically observed via invasive electroencephalography recordings. Moreover, the goodness of fit of the model was associated with the outcome of epilepsy surgery. We used the model to generate alternative hypothesis of the seizure seed regions, and to simulate the effects of the resective surgery that each patient had undergone, and found indications that larger effects of virtual resections in the model may be associated with seizure freedom after the actual surgery. These findings illustrate how individualized computational models may aid epilepsy surgery planning by identifying alternative seed regions and/or resection strategies, with the ultimate goal of improving surgery outcomes.

## INTRODUCTION

Epilepsy is a highly prevalent neurological disorder, affecting between 4 and 10 per 1,000 people worldwide (Banerjee, Filippi, & Hauser, 2009). About 1 out of 3 people who suffer from epilepsy do not respond to medication, that is, they present drug-resistant or refractory epilepsy (Kwan et al., 2010). In these cases, epilepsy surgery, consisting of the removal or disconnection of the necessary brain regions to stop seizure propagation—namely the *epileptogenic zone* (Lüders, Najm, Nair, Widdess-Walsh, & Bingman, 2006)—is the treatment of choice. Several conditions must be met for the surgery to proceed, including that a focal origin of the seizures can be found, and that the proposed surgery can be performed safely, that is, without unwanted side effects such as sensorimotor deficits, amnesia, or aphasia. Surgery outcomes vary greatly depending on epilepsy type, with seizure freedom attained for about two thirds of the patients 1 year after surgery (Englot et al., 2015). Although the majority of patients still experience a reduction in seizure frequency or intensity after surgery, even when the resection is not completely successful, side effects and cognitive complaints are also common, and can be difficult to predict accurately on an individual basis (Jobst & Cascino, 2015).

In recent years, several efforts have been made to improve the outcome of epilepsy surgery. One important conceptual leap forward is the notion of *epileptogenic networks* (Bartolomei et al., 2017), according to which even in case of focal epilepsy the *epileptogenic focus* should not be considered as solely responsible for seizure generation and propagation, but rather the existing brain network also plays a role in promoting (or inhibiting) the ictal state (Kramer & Cash, 2012; Smith & Schevon, 2016; Stam, 2014). Within this perspective it has been found that some of the characteristic properties of the healthy brain (such optimal routing, scale invariance) (Seguin, van den Heuvel, & Zalesky, 2018) are systematically affected by neurological diseases (Stam, 2014), including epilepsy (Besson et al., 2017; Centeno & Carmichael, 2014; Douw et al., 2010; Sinha, Peternell, et al., 2021; Sinha, Wang, et al., 2021). In particular, abnormalities are often found relating to the *brain network hubs*, which often suffer from targeted damage in patients with neurological disorders (Moreno, Gómez, & Pacheco, 2003; Stam, 2014). In the case of epilepsy, hubs may facilitate the propagation of epileptiform activity throughout the brain (Jin, Jeong, & Chung, 2015; Ramaraju et al., 2020), and several studies have pointed out the existence of *pathological hubs*, that is, abnormal, hyperconnected regions in the vicinity of the epileptic focus, which mediate seizure propagation (da Silva et al., 2020; Jin et al., 2015; Liao et al., 2010; Nissen et al., 2017).

Within the network perspective, the effect of surgery is no longer straightforward to predict: local changes in a network may have widespread effects, or be compensated by the remaining network (Hebbink, Meijer, Huiskamp, van Gils, & Leijten, 2017; Nissen et al., 2018). Moreover, the specific effect of a surgery will depend on the individual network configuration (Gerster et al., 2021), making it fundamental to consider patient-specific connectivity in order to tailor the surgery specifically to each patient. Network-based studies have found group-level differences between patients with good and bad surgical outcomes (da Silva et al., 2020; Nissen et al., 2018; Taylor et al., 2018), for instance Nissen et al. (2017) found that the removal of a pathological hub, or a region highly connected to it, was strongly associated with seizure freedom.

A data-driven manner to address this problem is by considering computational models of brain dynamics, which in general can be used to better understand brain function and its relation with the underlying structure (Sorrentino et al., 2021; Tewarie et al., 2019), for instance, in the healthy and disease states. Computational models of epilepsy surgery simulate in silico different resection strategies to help predict their impact before hand, with the goal of improving the planning of resective epilepsy surgery (Goodfellow et al., 2016; Hutchings et al., 2015; Jirsa et al., 2017; Laiou et al., 2019; Lopes et al., 2017; Nissen et al., 2021; Olmi, Petkoski, Guye, Bartolomei, & Jirsa, 2019; Proix, Bartolomei, Chauvel, Bernard, & Jirsa, 2014; Sinha et al., 2017; Taylor, Kaiser, & Dauwels, 2014). In order to tailor the resection strategy for each patient, and thus increase the chances of seizure freedom, the models are fitted to patient-specific data such as the underlying brain network connectivity (derived via different imaging techniques), stereotypical patterns of seizure propagation and clinical biomarkers of the suspected location of the epileptogenic focus. Once the models have been defined, they can be used to predict the outcome of surgery (Jirsa et al., 2017; Sinha et al., 2017), or to propose alternative resection strategies, for instance, in the case of a previous bad outcome or inoperable regions (Sinha et al., 2017), or with a smaller impact than the actual surgery (An, Bartolomei, Guye, & Jirsa, 2019; Millán et al., 2022; Nissen et al., 2021; Olmi et al., 2019).

Computational models of epilepsy surgery rely on the definition of a dynamical model of seizure generation and propagation. However, the specific mechanisms underlying seizure dynamics are not well known and likely not unique: epilepsy is a heterogeneous disorder, and, for instance, different seizure types can be identified according to their onset, spreading and recovery patterns (Lagarde et al., 2016; Perucca, Dubeau, & Gotman, 2014). Mathematically, six different stereotypical patterns of seizure dynamics have been distinguished from the modeling perspective (Jirsa, Stacey, Quilichini, Ivanov, & Bernard, 2014; Saggio et al., 2020; Wang et al., 2017). Thus, assumptions must be made in the modeling of seizure dynamics, and different levels of description, at different scales, are possible (Depannemaecker, Destexhe, Jirsa, & Bernard, 2021). Realistic models make use of highly detailed nonlinear dynamics (Junges, Lopes, Terry, & Goodfellow, 2019), such as population rate models (Liou et al., 2020) or neural mass models, which are preferred in recent literature to model the effect of resections (Hashemi et al., 2020), combined with one or several slow variables to account for the transition from normal to ictal activity (Jirsa et al., 2017; Proix et al., 2014). Within this perspective, several studies have tried to model seizure dynamics and predict the outcome of epilepsy surgery, with remarkable success at a group level: Sinha et al. (2017), using a dynamical model based on electroencephalography (EEG) connectivity to identify epileptogenic regions, found that the overlap between these regions and the resection area predicted surgery outcome with 81.3% accuracy. Proix, Bartolomei, Guye, and Jirsa (2017) found that their seizure model, the *epileptor model* (Jirsa et al., 2014), defined over magnetic resonance imaging (MRI) networks, could distinguish between good (Engel class I) and bad (Engel class III) outcomes. Further studies within this modeling framework also found a better match between the hypothesized epileptogenic zone and propagation zone (i.e., the first regions to which ictal activity propagates to) for seizure-free (SF) than nonseizure-free (NSF) patients (Makhalova et al., 2022; Vattikonda et al., 2021). One a virtual resection study Sip et al. (2021) found that the effect of the resection in the model correlated with surgical outcome, so that patients with Engel score I and II presented a significantly larger effect of virtual resections in the model. Similarly, Goodfellow et al. (2016) also found significant differences in the model prediction for Engel class I and class IV patients, using an electrocorticogram modeling framework.

Detailed models of ictal activity, however, come at a high cost: several parameters need to be adjusted beforehand, with unavoidable arbitrary choices. This complicates the setting of the model parameters and either large quantities of data are needed or several assumptions must be made. Similarly, the estimation of alternative resection strategies also suffers from degeneracy issues that worsen as the degrees of freedom are increased. Restrictions are usually imposed, such as considering only resections ipsilateral to the hypothesized seizure onset zone or viable resection, for example, avoiding eloquent cortex or nonfocal resections. Due to the risk of overfitting, generalizing the results to new datasets becomes troublesome. In order to address this problem, in-depth studies to characterize the dynamical properties of the models, and the interplay between network structure and emergent dynamics, are needed (Courtiol, Guye, Bartolomei, Petkoski, & Jirsa, 2020; El Houssaini, Bernard, & Jirsa, 2020; Sip, Guye, Bartolomei, & Jirsa, 2022), often in combination with elaborate modeling optimization frameworks, such as Bayesian inference (Hashemi et al., 2020, 2021; Sip et al., 2021; Vattikonda et al., 2021) or deep learning (Hashemi et al., 2022). Another possibility to circumvent these issues is by considering simpler, abstract models that focus only on the behavior of interest: the propagation of ictal activity throughout the brain (Millán et al., 2022; Nissen et al., 2021), typically accounted for by a slow permittivity in higher dimensional seizure models (Hashemi et al., 2020; Sip et al., 2021). Conceptually, this process is equivalent to other spreading processes on networks, a problem that has been well characterized by means of *epidemic spreading models* (Pastor-Satorras, Castellano, Van Mieghem, & Vespignani, 2015). Epidemic spreading models simulate the propagation of an agent from some given location on a network to other connected areas, a basic phenomenon appearing in a multitude of systems. In the case of brain dynamics, such models have been used to study the spreading of pathological proteins on brain networks (Peraza et al., 2019; Schoonhoven et al., 2022), or the relation between brain structure and function (Stam et al., 2016). Due to their ubiquity and relative mathematical simplicity, epidemic spreading models are supported by a wealth of mathematical background characterizing the emergent dynamics in relation to different properties of the underlying network. This information can later be useful for clinical applications, for example, general rules for spreading phenomena on complex networks that can be applied to understanding seizure propagation.

In previous studies we considered epidemic spreading models as the basis for seizure propagation over the brain, without trying to mimic the complicated biophysical mechanisms involved in the process (Millán et al., 2022; Nissen et al., 2021). Within this framework, we found that epidemic spreading models fitted with patient-specific data could reproduce the stereotypical patterns of seizure propagation on patient-specific brain networks, individually for each patient. Moreover, by taking into account this patient-specific connectivity, alternative or smaller resections could be found with the model, which we hypothesized could lead to fewer side effects with the same outcome, in terms of seizure reduction (Millán et al., 2022; Nissen et al., 2021).

Here we consider an epidemic spreading model to generate individualized seizure propagation models that are based on the patient-specific MEG connectivity and seizure propagation pathways as derived from invasive EEG recordings. We considered a group of 15 epilepsy patients who underwent epilepsy surgery, and for whom the surgical outcome at least 1 year after surgery was known. This framework generalizes on previous studies by our group (Millán et al., 2022; Nissen et al., 2021) by including a recovery mechanism in the spreading model, allowing the return to the healthy (postictal) state, so that seizures may remain local (i.e., if the affected regions recover before propagating the ictal state to distant regions) or generalize. In this study we also take a step further by considering how the present model can be used to generate alternative hypotheses on the seizure onset zone and test different resection strategies, potentially before the surgery has taken place. We discuss the challenges associated to the model fitting, even in this simple scenario, and associated risks. We also present for the first time a population model that integrates seizure propagation data from all patients but can still be individualized and applied to patients without SEEG recordings. We discuss how this approach can help reducing the overfitting risk and noise effects, and how it may increase the generizability and clinical application of epilepsy surgery models.

## RESULTS

**Figure 1.**
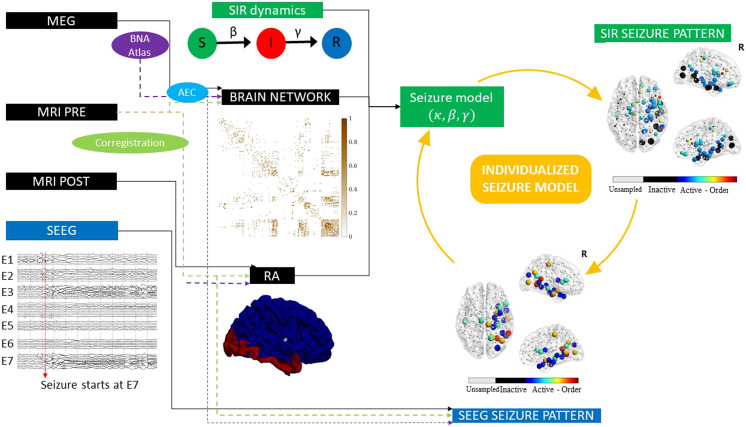
Sketch of the methodology followed in this study. The Spreading-Infected-Recovered (SIR) model was used to simulate seizure propagation. As the backbone for the model dynamics, we used the patient-specific MEG network, and the seed regions were initially defined as the resection area (RA), which was reconstructed from the pre- and postsurgery MRIs. By analyzing the seizures generated by the model, we derived the *SIR seizure pattern*, describing seizure propagation in the model. This was compared to the *SEEG seizure pattern* as derived from SEEG recordings of ictal activity. The seizure propagation patterns describe the activation order the active (i.e., infected, in the ictal state) and sampled (by the SEEG electrodes) regions of interest. Comparison between the model and the data as described in the main text allowed us to fit the model parameters to the SEEG pattern and create an *individualized seizure propagation model* for each patient.

The individualized seizure propagation models were based on an epidemic spreading model—the Susceptible-Infected-Recovered or SIR model—equipped with patient-specific data, as depicted in Figure 1. A total of 15 patients (9 females) were included in the study, 11 of whom were seizure free (SF) 1 year after surgery (Engel class 1A, see Table 1 for the patient details). This patient cohort was partially used in our previous study (Millán et al., 2022). For more details see the Methods section.

**Table 1.** Patient data

Case	Sex	Resection area	*S*_RA_	Engel score	#E	#ECP	*N_SR_*
P1	F	R frontal	4	1A	13	128	47
P2	F	R temporal, occipital	13	1A	14	142	50
P3	F	L temporal, occipital	5	1A	15	144	53
P4	M	R temporal	13	1A	13	126	49
P5	F	R temporal	10	1A	11	109	42
**P6**	**F**	**R lat. temporal**	**5**	**2A**	**9**	**99**	**40**
P7	F	L temporal	5	1A	11	110	44
P8	F	L parietal	4	1A	10	104	37
P9	M	R post. lat. temp., post. ins., post. par.	3	1A	12	102	38
**P10**	**F**	**R temporal**	**13**	**2D**	**11**	**114**	**45**
**P11**	**F**	**L frontal**	**4**	**2C**	**13**	**117**	**47**
P12	M	L frontal	6	1A	12	124	40
P13	M	L temporal	5	1A	12	106	30
**P14**	**F**	**L temporal**	**6**	**3A**	**15**	**194**	**60**
P15	M	R temporal	12	1A	10	107	32

*Note*. NSF cases are indicated by boldface. Ep. = Epilepsy, y = years, *S*_RA_ = number of resected ROIs, #E = number of intracranial electrodes, #ECP = total number of electrode contact points, *N*_*SR*_ = number of brain regions sampled by the SEEG electrodes. F = female, M = male, R = right, L = left.

### Seizure Propagation as an Epidemic Spreading Process

Seizure propagation was modeled using the SIR model such that the susceptible (S), infected (I), and recovered (R) states accounted, respectively, for the healthy (preictal), ictal, and healthy (postictal) states. The SIR model describes the spreading of an epidemic process on a network from a set of seed regions to the other nodes, and it has been applied in a multitude of scenarios involving spreading phenomena. The emerging behavior of the system under this dynamics is well characterized in relation to the underlying network structure (Barrat, Barthelemy, & Vespignani, 2008; Pastor-Satorras et al., 2015). In this scenario, the model does not try to mimic the detailed biophysical processes involved in seizure generation and propagation, instead it is used here as an abstraction that includes only the most relevant features of seizure propagation (Millán et al., 2022; Nissen et al., 2021; Pastor-Satorras et al., 2015; Sip et al., 2021). The model is characterized by two control parameters, the global spreading rate *β* characterizing the probability of spreading of the infected state, and the recovery rate *γ* characterizing the recovery probability of each infected node.

The model was simulated on top of the patient’s brain network reconstructed from resting-state MEG recordings using the Brainnetome Atlas (246 nodes). Each region of interest (ROI) was represented via a node *i* in the network, and each connection via a link (*i*, *j*), with the weight *w*_*ij*_ of link (*i*, *j*) indicating the strength of the coupling between ROIs *i* and *j*. The weight distribution affected the propagation pattern as *w*_*ij*_ modulated locally the spreading rate: the probability that an infected node *i* infected a neighbour *j* was given by *βw*_*ij*_. Thus, strongly connected neighbours were more likely to propagate the infected state. As coupling metric we considered the uncorrected amplitude envelope correlation (AEC). AEC-MEG networks include both short- and long-range functional connections, combining in one network aspects of structural and functional connectivity. In a previous study (Millán et al., 2022), we validated the use of AEC-MEG networks as the backbone for seizure propagation, and we found that AEC-MEG networks strongly correlated with modeled structural networks (as given by the exponential distance rule; see the Methods section for details). The networks were thresholded (but not binearized), with the link density *κ* acting as the third control parameter of the model. An exemplary case of the final weight matrix is shown in Figure 1.

**Figure 2.**
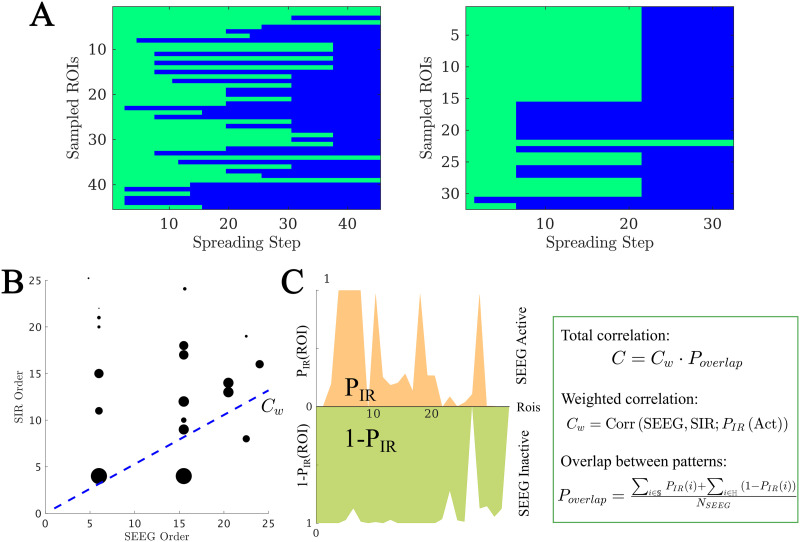
(A) Exemplary SEEG seizure propagation patterns corresponding to cases 15 and 9, indicating the state of each ROI (preictal or ictal) at each time step. ROIs in the preictal state are shown in green, whereas ictal and postictal ROIs are shown in blue. We only show ROIs sampled by the SEEG contact points. The first case shows a “linear” pattern, where ROIs are infected roughly one by one, whereas the second shows a “bulky” activation pattern where several ROIs get infected at the same time. (B and C) Total correlation *C* between the SIR and SEEG seizure patterns. First, the set of active (i.e., infected) ROIs in both patterns was identified, and the weighted correlation *C*_*w*_ between the activation orders was calculated (panel B, blue line). As correlation weights we used the probability that the ROI *i* was infected in the SIR pattern, *P*_*IR*_(*i*), depicted in the figure by the size of the black circles. Then, to control for the extension of the seizure in both patterns, we computed the weighted overlap between the active and inactive regions (panel C), *P*_*overlap*_. *P*_*IR*_(*i*) is the probability that the ROI *i* becomes infected during the spreading process. Conversely, 1 − *P*_*IR*_(*i*) is the probability that it does not become infected. 𝕊 and ℍ stand, respectively, for the sets of ROIs that are infected (i.e., in the seizure state) and not infected (i.e., in the healthy state) in the SEEG pattern. The total correlation was then defined as *C* = *C*_*w*_*P*_*overlap*_. For more details see section Seizure Propagation Model. The seizure propagation patterns corresponding to this data are shown in Figure 1 under “SIR Seizure Pattern” and “SEEG Seizure Pattern,” respectively.

The seizure propagation model was adapted individually for each patient by fitting the simulated propagation patterns to patient-specific seizure propagation data derived from SEEG recordings, and by using the resection area (RA) as the seed of epidemic spreading (see Seizure Propagation Model for more details). In order to do this, two seizure propagation patterns were constructed, the SIR and the SEEG seizure patterns, depicting, respectively, the activation order of the sampled ROIs in the SIR- and SEEG-derived seizures. An exemplary case is shown in Figure 1. The total correlation between the two patterns, *C*, as defined by Equation 6 in the Methods, and illustrated in Figure 2B (for the same exemplary case as in Figure 1), was used as the *goodness of fit* of the model (see Seizure Propagation Model).

This framework builds on our previous works (Millán et al., 2022; Nissen et al., 2021) but has significant key differences in methodology (which are specified in detail in the Methods section) and application. We already introduced the use of the SIR model to improve epilepsy surgery in Nissen et al. (2021), and in Millán et al. (2022) we introduced the use of AEC-MEG networks as backbone for seizure propagation, and the procedure to fit the model parameters to the SEEG data, although the seizure model was based on the SI dynamics. Here we have combined the two frameworks to fit the SIR parameters to the SEEG data, which allows for a more accurate depiction of seizure propagation, as it allows for the recovery of individual regions before the end of the seizure. As a consequence, however, the fitting method differs from the one in Millán et al. (2022), and cannot be compared directly. Moreover, both in Millán et al. (2022) and Nissen et al. (2021) we performed an optimization study to find alternative resection strategies with a smaller extension than the actual surgery, assuming that the resection area was the epidemic seed. However, this is an oversimplification that is only expected to hold for seizure-free patients. Indeed, for nonseizure-free patients the two could differ completely, and even for seizure-free patients the resection may have spared epileptogenic tissue (just not enough to lead to seizures) or removed healthy tissue. Therefore, in this study we propose a different methodology by identifying alternative seed regions based on the SEEG recordings and testing the effect of the actual surgery in the model. In the future, this approach could be extended to patients prior to the surgery, and so gain information on the expected effect of a proposed resection strategy.

### Individualized Seizure Propagation Models

**Figure 3.**
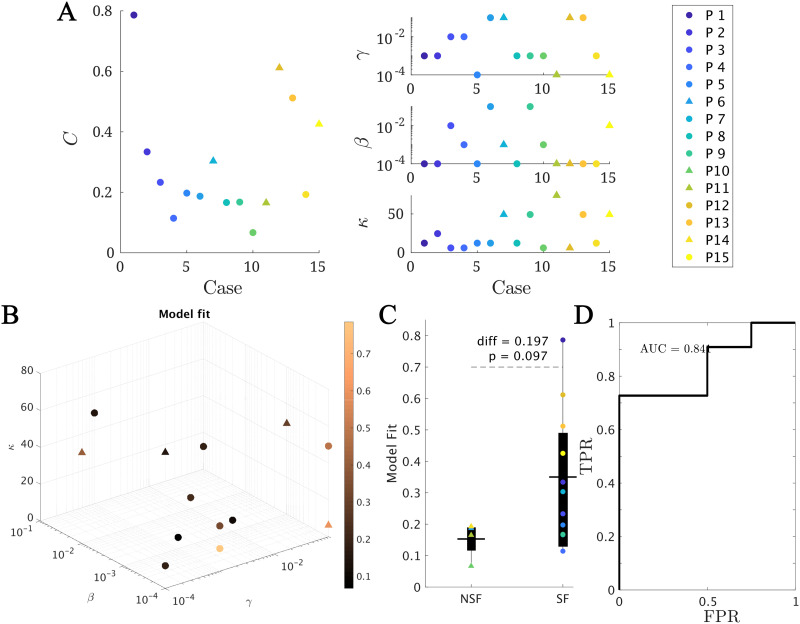
Model fitting results. (A and B) The best fit found for each patient. In panel A, the main (left) panel shows the resulting model fit (as given by the total correlation *C*) and the three small panels (right) show the corresponding fit parameters (*κ*, *β*, *γ*). Each patient is shown in a different color as indicated by the main legend, and SF and NSF patients are, respectively, indicated by circles and triangles. In panel B the model parameters (*κ*, *β*, *γ*) form the three axes of the plot and the color code indicates the goodness of fit *C*. We note that some points overlap. (C) Group comparison of the goodness of fit, for the NSF and SF groups (unpaired *t* test). Each color represents a different patient, as indicated by the main legend. The solid lines on each box indicate the mean values. In panels A and B, SF (NSF) patients are indicated by circles (triangles). (D) Receiver operating characteristic (ROC) curve corresponding to the group classification according to the goodness of fit. A positive result was defined as a good outcome (SF). FPR indicates the false positive rate (NSF patients classified as SF), and TPR the true positive rate (SF patients classified as SF).

Within this framework, we found the set of parameters (*κ*, *β*, *γ*) yielding the best model fit *C* for each patient (see Methods for details and Figure 3A for the fit results). On average, we obtained a model fit of *C* = 0.30 (with standard deviation std_*C*_ = 0.20). The model provided a (not significantly) better fit for the SF (*C*_*SF*_ = 0.35) than NSF (*C*_NSF_ = 0.15) patient groups (*C*_*SF*_ − *C*_NSF_ = 0.20, *t*(13) = 1.79, *p* = 0.097, unpaired *t* test), as shown in Figure 3B. A receiver-operating characteristic (ROC) classification analysis based on the goodness of fit returned a good classification result with an area under the curve (AUC) of AUC = 0.841. There were no significant differences in the fit parameters between the groups (average ± standard deviation: *κ* = 25 ± 22, *β* = 0.02 ± 0.03, *γ* = 0.03 ± 0.04).

### Population Model

**Figure 4.**
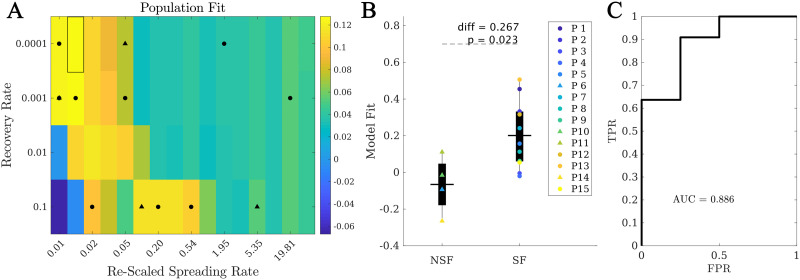
Population model. (A) Phase diagram showing the population fit $\bar{C}$ (*βκ*, *γ*), where *βκ* is the rescaled spreading rate. Black markers indicate the location of the best individual fits in this diagram, with circles (triangles) corresponding to SF (NSF) patients. The highlighted rectangle indicates the best fit. (B) Comparison between the population fit for the SF and NSF groups. Solid lines indicate the mean values for each group. (C) ROC classification analysis between the SF and NSF groups, with an AUC = 0.886.

The population model $\bar{C}$(*β*, *γ*, *κ*) was defined by measuring the average fit across patients using the algebraic population average of the model fit for each parameter configuration: $\bar{C}$(*β*, *γ*, *κ*) = $N_{\text{pats}}^{-1}$ ∑_*pat*_ *C*_*pat*_(*β*, *γ*, *κ*). As with the individual model, the local spreading rates were defined for each patient based on their individual AEC-MEG network, and the seed was based on their individual resection area. Thus, the only difference between the population model and the individual models is in the global parameters (*β*, *γ*, *κ*): for the individual model we used the parameters leading to the best fit for each patient, whereas for the population model we used the parameters leading to the best fit over the population. The resulting fit diagram is shown (Figure 4), where for visualization purposes we have combined *β* and *γ* into the rescaled spreading rate, defined as *βκ*, to quantify in a single quantity the main parameters controlling the speed of spreading. The original fit diagrams in terms of *γ*, *β*, and *κ* can be seen in the Supporting Information (Figure S2). In the rescaled 2D representation, the resulting fit diagram resembles a familiar phase transition diagram, with an interface of high goodness of fit (yellow regions) corresponding to a roughly constant spreading-to-recovery ratio *βκ*/*γ* = const. Most individual best fits (black markers) fell within this region, although there was large variability among the individual results (in fact, we found low signal to noise ratios of approximately one fifth as shown in the Supporting Information Figure S2 and S3).

Within the population model, the best fit was $\bar{C}$ = 0.13 ± 0.19, corresponding to *β* = 10^−4^, *κ* = 24.6, *γ* = 10^−4^ (highlighted rectangle in Figure 4). Remarkably, when considering the fit results for each patient at the optimal population point, we found that the SF group ($\bar{C}$(*SF*) = 0.20 ± 0.18) presented a significantly better fit than the NSF group ($\bar{C}$(NSF) = −0.07 ± 0.16, *C*(*SF*) − *C*(*NSF*) = 0.27, *t*(13) = 2.59, *p* = 0.02), as shown in Figure 4B. Moreover, the ROC classification analysis in this case also provided a good classification (AUC = 0.886) between the SF and NSF groups (see Figure 4C). Finally, we also compared the population model as defined independently for the SF and NSF groups (Supporting Information Figure S2 and S3). The NSF fit map presented generally lower values and more narrow areas of high goodness of fit. The optimal fit differed slightly for each group (SF: *γ* = 10^−3^, *β* = 10^−4^, *κ* = 24.6, *C* = 0.203; NSF: *γ* = 10^−3^, *β* = 10^−4^, *κ* = 12.3, *C* = 0.056), and the average goodness of fit was larger for the SF group than the NSF group (diff = 0.147, *t*(13) = 1.46, *p* = 0.17), but not significantly.

### Alternative Seizure Onset Zones

Once the model was fitted to the patient-specific seizure propagation patterns, we estimated the likelihood of each individual ROI acting as the seizure onset zone (SOZ). We defined this as the total correlation metric when ROI *R* was used as the single seed for the SIR dynamics, *C*_*R*_. For this analysis we considered the individualized spreading models (i.e., fitted individually for each patient) as they provided a better characterization of the individual SEEG seizure propagation patterns. We did not fit the model parameters again for each individual seed, which may have caused suboptimal goodness of fit of the individual seeds. Considering different model parameters for each seed would also poss a problem, however, as one would not expect the global properties of the system to change depending on the seed region, as different seeds could be active for different seizures. Moreover, increasing the dimensionality of the fitting problem in such manner could easily lead to unreliable results that lack specificity. Thus, we decided to fix the model parameters to limit the associated parameter degeneracy and computational demands. In order to maintain the initial spreading level for all seeds, the spreading rate was the adapted by the out-connectivity of the seed, as discussed in the Methods section.

**Figure 5.**
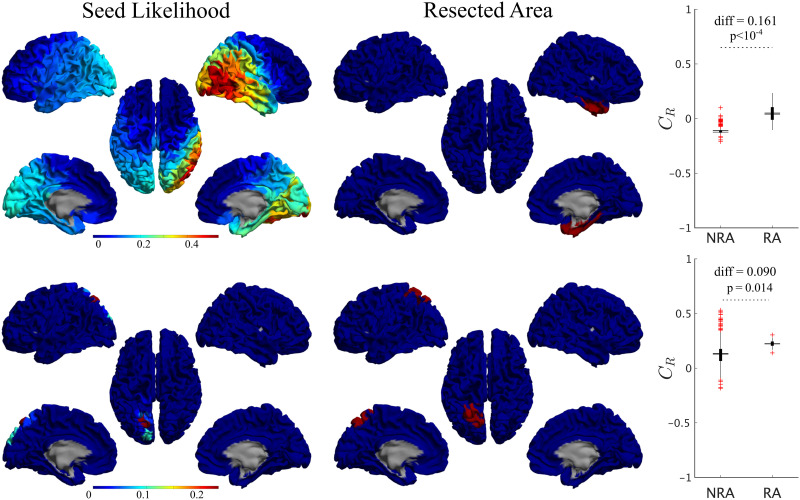
Seed-likelihood maps for two representative cases. Left panels show the seed likelihood of each ROI, *C*_*R*_, for two exemplary cases (cases 13 and 15, respectively, from top to bottom), whereas the middle panels show the corresponding resection areas in red. The right panels indicate the comparison between RA and non-RA (NRA) ROIs, for these two cases (unpaired *t* test). The solid lines stand for the mean values. Red pluses mark outliers (1.5 times over the interquartile range).

For all patients, single seeds could be found that provided a good approximation to the seizure propagation pathways, that is, with high *C*_*R*_ values, as shown in Figure 5 (left panels) for two exemplary cases. The seed likelihood maps depicted different degrees of localization and sparseness for different patients, as well as different degrees of overlap with the RA (also shown in Figure 5 for comparison purposes, middle panels). From visual inspection, the RA tended to appear in regions with relatively high *C*_*R*_, but did not include the maximum. In order to test whether RA ROIs tended to have higher *C*_*R*_ than non-RA ROIs, we compared the seed likelihood for the two ROI sets, for each patient, as shown in the right-side panels in Figure 5. In these two exemplary cases, RA ROIs were significantly more likely to be the seed than non-RA ROIs. However, this was the case only for 7 out of 15 cases, of which 1 was NSF. For the remaining 8 cases, no significant difference between the groups was found (see Supporting Information Table S1).

**Figure 6.**
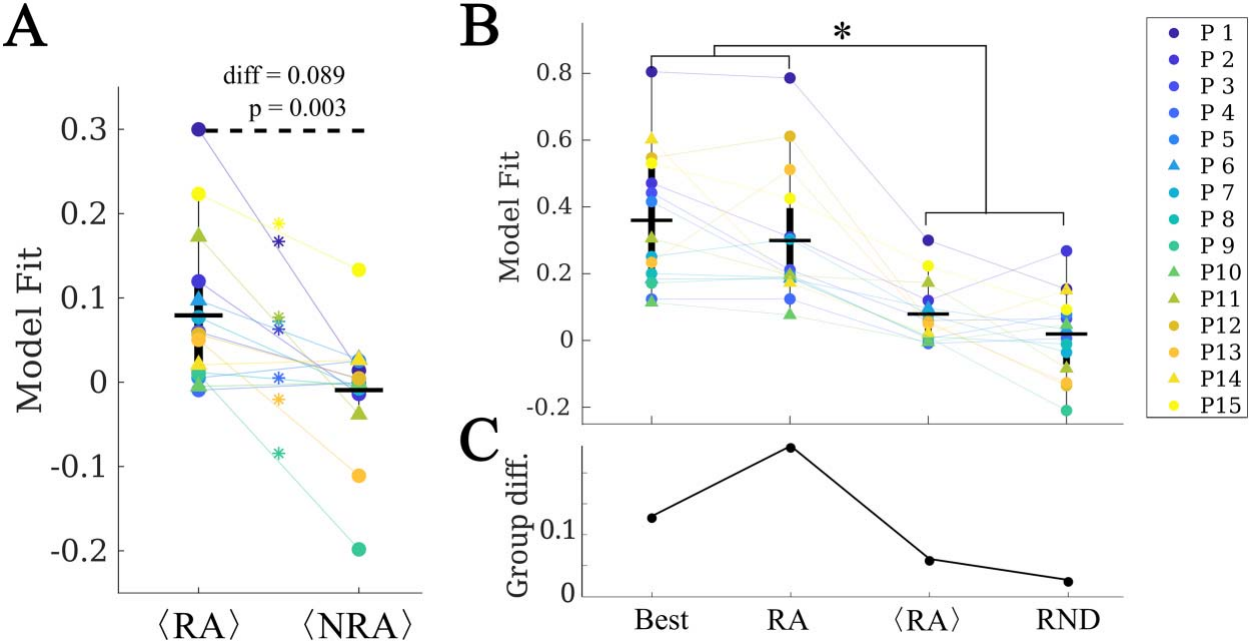
Analysis of alternative seeds. (A) RA regions had a significantly higher model fit (given by the total correlation when considering each ROI as the single epidemic seed, *C*_*R*_) than non-RA regions (NRA, paired *t* test) on average. Stars indicate a significance difference at the patient level. (B) Effect of the seed choice on the model fit. *Best* stands for the best single seed, *RA* for using the whole RA, 〈*RA*〉 for the average of the RA ROIs single seed fits, and *RND* for random seeds of the same size as the RA. (C) Group difference (SF vs. NSF) found for each of the seed fits. The two groups only differed significantly when using the RA as seed.

At a group level, we found that RA ROIs were on average more likely to be the seed than non-RA ROIs, as shown in Figure 6A (*C*_RA_ − *C*_NRA_ = 0.077, *p* = 0.016, *t*(14) = 2.74, paired *t* test). However, the ROI with the maximum likelihood, *C*_best_, did not belong to the RA for any case (see, for instance, the two exemplary cases shown in Figure 5). Thus, the most likely single seeds were close to the RA, but did not belong to it. Despite the individual best seeds (Best) performing better than the RA, the difference was not significant. Moreover, both the Best and RA seeds performed better than the averaged individual RA ROIs, 〈*RA*〉, and than random seeds of the same size as the RA, *RND* (see Supporting Information for details of the comparisons).

No difference in the average seed likelihood of the RA was found between the SF and NSF patients (*C*_RA,SF_ − *C*_RA,NSF_ = −0.006, *t*(13) = −0.09, *p* = 0.93, unpaired *t* test), or in the maximum single seed likelihood, *C*_max_ (*C*_best,SF_ − *C*_best,NSF_ = 0.08, *t*(14) = 0.6, *p* = 0.5), as shown in Figure 6C. Moreover, the difference between the SF and NSF groups also vanished when considering random seeds (*C*_RND,SF_ − *C*_RND,NSF_ = −0.02, *t*(14) = −0.3, *p* = 0.8, Figure 6).

### Virtual Resection Analysis

We performed a virtual resection analysis to simulate the effect of the surgery for each patient. Given that the actual seizure onset zone is not known, here we considered the optimal seeds in terms of the goodness of fit (as derived via a stepwise recursive procedure), with increasing seed sizes. Due to the degeneracy of the model, seeds of different sizes can have similar goodness of fit, and the problem of finding optimal seeds of different sizes is of divergent complexity. Therefore, we manually set a cutoff of five ROI seeds to compare all patients under the same basis. By repeating the analyses for different sizes, we also tested whether the results are robust to the exact seed definition. The method to derive the seed regions and perform virtual resection is explained in detail in the Methods section.

**Figure 7.**
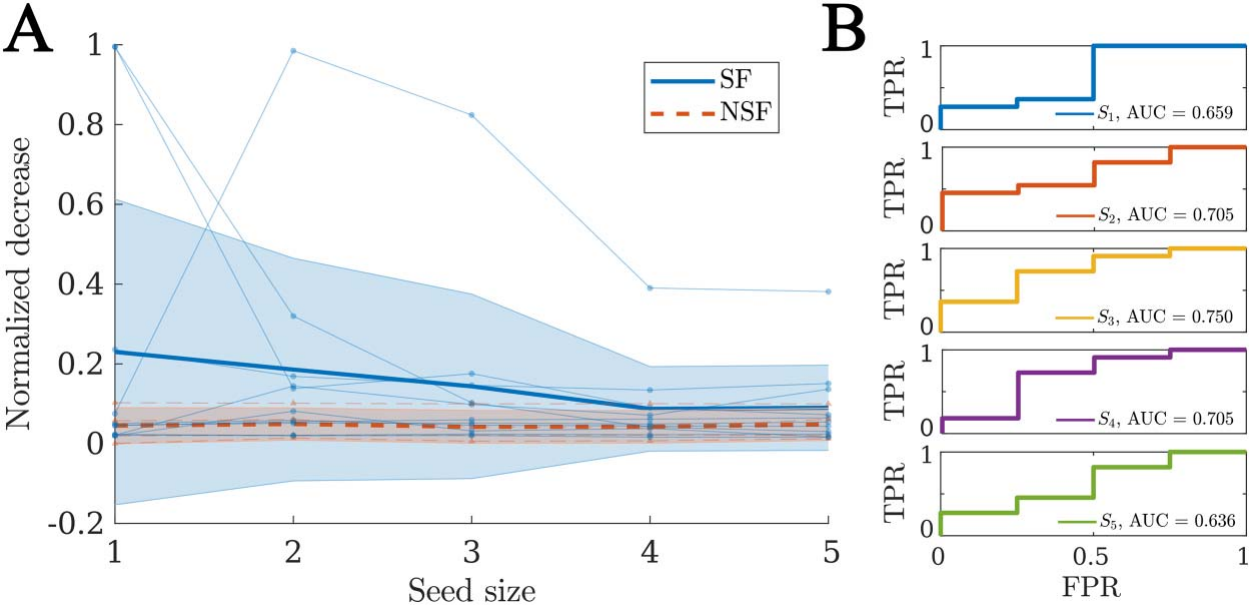
Virtual resection analysis. (A) Normalized decrease in spreading after disconnection of the resection area *δ*_*R*_ for seeds of increasing sizes. Each data point indicates an individual patient, blue circles stand for SF patients, and red triangles for NSF patients. The thick lines indicate the average values for each group, respectively, solid blue lines for the SF group and dashed red lines for the NSF group. The shaded areas indicate the uncertainty as given by the standard deviation. (B) ROC classification analysis for SF versus NSF outcome, for each considered seed size as indicated in the legends.

For each patient, there was a significant decrease in seizure propagation with the surgery for all considered seed sizes, as given by the normalized decrease in spreading *δ*_*R*_. We found that the SF group presented larger *δ*_*R*_ (see Figure 7A) for all considered sizes, but the difference was not significant in any case (see Supporting Information Figure S2 for details of the comparisons). Finally, when considering the normalized decrease as a classification metric for SF versus NSF patients we found AUC values between 0.636 and 0.750 (average = 0.691), as shown in Figure 7B.

In order to understand what defines the effect of the resection, we computed the correlation between the normalized decrease in logarithmic scale log (*δ*_*R*_) and different dynamical and network properties (see Table 2) that characterize the network structure before and after the resection, as well as the effect of the resection. We also considered a model-based metric, the spreading ratio *βκ*/*γ*, which combines all three network parameters in one. We also considered the network-based properties of (i) the resection area before the resection, (ii) the seed before and after the resection, and (iii) the change Δ in the seed status due to the resection. The considered network variables are the size *S* (i.e., number of nodes), out-connectivity *E* (number of links from the considered node set to the rest of the network), betweenness centrality *BC*, clustering *c*, and efficiency *F*. The clustering and efficiency can also be defined globally so we also considered these properties (before the resection, after the resection, and the change due to the resection) at the global network level. These variables are described in Table 3, and specific definitions are included in the Methods section.

**Table 2.** Relation between the effect of virtual resections of the RA (as given by the normalized decrease in spreading after the resection) and the properties of the model, baseline, and postresection networks

Baseline	Effect	*r* ^2^	*p*	Resection	Effect	*r* ^2^	*p*
*βκ*/*γ*	+	0.09	**0.01**	*E*_seed_	−	0.10	**0.006**
*S*_RA_	+	0.33	**8 · 10^−8^**	Δ*E*_seed_	+	0.06	**0.04**
*S*_seed_	−	< 10^−2^	0.7	BC_seed_	−	0.11	**0.004**
*E*_RA_	+	0.11	**0.004**	ΔBC_seed_	+	0.15	**5 · 10^−4^**
*E*_seed_	−	0.02	0.2	*c*_seed_	−	0.21	**3 · 10^−5^**
BC_RA_	+	0.08	**0.01**	*c*_Netw_	−	< 10^−2^	0.5
BC_seed_	−	0.05	0.06	Δ*c*_seed_	+	0.27	**2 · 10^−5^**
*c*_RA_	+	0.003	0.6	Δ*c*_Netw_	+	0.08	**0.01**
*c*_seed_	−	0.11	**0.004**	*F*_seed_	−	0.21	**3 · 10^−5^**
*c*_Netw_	−	0.002	0.7	*F*_Netw_	−	< 10^−2^	0.8
*F*_RA_	+	< 10^−2^	0.8	Δ*F*_seed_	+	0.29	**7 · 10^−7^**
*F*_seed_	−	0.02	0.2	Δ*F*_Netw_	+	0.11	**0.003**
*F*_Netw_	−	2 · 10^−5^	0.9				

*Note*. As model parameter we consider the spreading-to-recovery ratio *βκ*/*γ*, which combines the three model parameters in one. The preresection network was characterized by the size *S*, the out-connectivity *E*, the betweenness centrality BC, the clustering *c*, and the efficiency *F* of the RA and the seed, and by the average clustering coefficient and efficiency of the network. The resected network (i.e., the network after the virtual resection of the RA was performed) was characterized by the new out-connectivity, BC, clustering, and efficiency of the seed, by the network-averaged clustering and efficiency, and by their respective decreases due to the virtual resection, that is, Δ*X* = *X*(baseline) − *X*(resection). Significant effects (*p* > 0.05) are indicated by bold font in the *p* value.

**Table 3.** Summary of acronyms (left) and of model and network variables (right)

Acronym	Definition	Variable	Definition
MEG	Magnetoencephalography	*β*	Global spreading rate
EEG	Electroencephalography	*γ*	Global recovery rate
MRI	Magnetic resonance imaging	*κ*	Mean degree
DTI	Diffusion tracktography imaging	*C*	Goodness of fit
ROI	Region of interest	*C*_*R*_	Seed likelihood
AEC	Amplitude envelope coupling	*IR*	Total spreading
BNA	BrainNetome Atlas	*δ_R_*	Normalized decrease in spreading
RA	Resection area	*BC*_*X*_	Betweenness centrality of *X*
SF	Seizure free	*c*_*X*_	Clustering of *X*
NSF	Nonseizure free	*F*_*X*_	Efficiency of *X*
SOZ	Seizure onset zone	*E*_*X*_	Out-connectivity of *X*
SIR	Susceptible-Infected-Recovered	*S*_*X*_	Size of *X*
		Δ*Y*_*X*_	Change in *Y*_*X*_ due to the resection

We found that the largest amount of variance was explained by the size of the resection *S*_RA_ (see Table 2), with larger resections leading to a larger effect of the virtual resection, as one might expect. The centrality of the RA (given by the out-connectivity *E*_RA_ and betweenness centrality BC_RA_) also correlated significantly with the effect of the resection, although the effect was weaker. However, the efficiency and clustering of the RA did not correlate with the effect of the resection.

Remarkably, the baseline centrality properties of the seed regions (i.e., size *S*_seed_, out-connectivity *E*_seed_, BC, BC_seed_, and efficiency *F*_seed_) did not show significant effects except for the clustering *c*_seed_. On the other hand, the properties of the seed *after* the resection did show a significant negative correlation with the effect of the resection on spreading. The structural effect of the resection, given by the decrease in centrality (out-connectivity Δ*E*_seed_, BC ΔBC_seed_, and efficiency Δ*F*_seed_) and clustering Δ*c*_seed_ of the seed was significantly and positively correlated with the effect of the resection on decreasing seizure propagation in the model. Overall, the clustering and efficiency of the seed after the resection, and their decrease due to the resection, showed the strongest associations with the decrease in spreading (after the size of the resection area). Of the network properties, only the decrease of efficiency of the network showed a positive correlation with the effect of the resection. The model parameters also played a role in the effect of the virtual resections, with larger spreading-to-recovery ratios associated with larger effects of the surgery.

**Figure 8.**
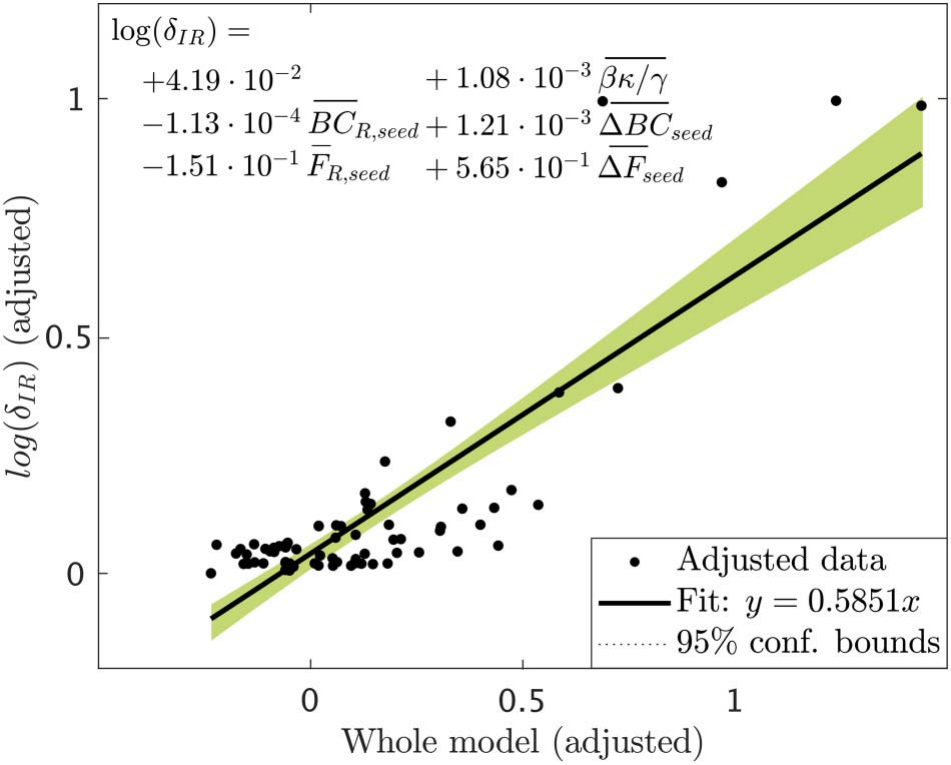
Added variable plot (partial regression leverage plot) of the linear regression model resulted from the stepwise regression analysis. Data points indicate the adjusted response values against the adjusted predictor variable values, the solid line indicates the adjusted linear fit, and the shaded areas the 95% confidence intervals. The bars over the variable names indicate adjusted variables. The statistical details of the fit are: number of observations = 75, degrees of freedom *df* = 69, root mean squared error *rmse* = 0.118, determination coefficient *r*^2^ = 0.715, Adjusted *r*^2^ = 0.694, F-statistic vs. constant model = 34.5, *p* = 1.6 · 10^−17^.

The variables considered in the previous analysis are not independent: the different centrality metrics are related, the properties of the resection area impact the structural effect of the resection, and the change of seed status is related to its status before and after the resection, for instance. In order to identify the most relevant properties determining the effect of the resection, we performed a stepwise linear regression analysis. As dependent variable we considered the normalized effect of the resection, in logarithmic scale, log (*δ*_*IR*_(*i*, seed_*j*_)), for each patient *i* and seed *j*. The resulting (adjusted) model is shown in Figure 8. We found that only five variables survived: the spreading ratio *βκ*/*γ* and the BC and efficiency of the seed in the resected network, BC_R,seed_ and *F*_R,seed_ and their decrease due to the resection, ΔBC_seed_ and ΔBC_seed_. The partial effect of all other variables was not significant once these five metrics were included in the model. The adjusted model achieved a goodness of fit *r*^2^ = 0.715 (see Figure 8).

These analyses indicate that the effect of virtual resections in the model is predominantly characterized by the size of the RA, as one might expect, but also by the centrality properties of the seed in relation to the RA. That is, both the hub status of the seed *after* the resection, and the decrease in hub status due to the resection were important for the decrease in spreading, but not the initial hub status per se.

## DISCUSSION

We have defined a computational framework to simulate seizure propagation and epilepsy surgery based on epidemic spreading models that integrate patient-specific data. A model was built for each patient based on their individual AEC-MEG brain network to combine structural and functional connectivity, and the propagation of ictal activity over the brain was modeled by means of a simple epidemic spreading model. The model was further individualized for each patient by fitting the main parameters, namely the spreading and recovery rates, and the network density, to the patient-specific seizure propagation patterns, as derived from SEEG data. We found that the model reproduced the main aspects of seizure propagation for all patients, indicating that these simple spreading rules are enough to encode the basic aspects of seizure propagation. Once fitted for each patient, the model can be used to generate alternative hypotheses about the seizure onset zone, or to test the effect of resection strategies, as we have illustrated in this study.

This study confirmed our previous findings in Millán et al. (2022) in that epidemic spreading models capture the basic aspects of seizure propagation. Here we have considered a slightly more detailed model that includes a recovery mechanism, and have found that this allows us to classify the patients according to surgical outcome. The SIR model allowed us to tune the spreading-to-recovery ratio for each patient, according to the recorded SEEG seizures, which, as we have found, strongly influences the effect of a resection in the model. Moreover, due to the recovery mechanism the simulated seizures may end before generalizing to the whole network, allowing us to measure the effect of virtual resections in the model more directly (whereas before an arbitrary point at which to measure propagation was needed (Millán et al., 2022; Nissen et al., 2021)). Finally, as opposed to our previous studies (Millán et al., 2022; Nissen et al., 2021) where we performed an optimization of virtual resections, with the seed set equal to the resection area, to find alternative resection strategies, here we tested the effect of the actual surgery on the model, using seed regions as identified by the model. In this manner we tested how the model could be implemented in the clinic to estimate the effect of a proposed resection strategy. The two approaches could be combined in future studies to identify alternative seed regions and optimal resection strategies with the model.

Epidemic spreading models capture the basic mechanisms of processes that propagate on networked systems and are supported by a well-grounded mathematical and computational framework (Moretti & Muñoz, 2013; Pastor-Satorras et al., 2015) that we can use to our advantage in the context of epilepsy surgery. For example, the fundamental role of hubs on surgical outcomes is expected from the perspective of epidemic spreading, as the epidemic threshold is known to vanish for networks with a scale-free degree distribution (and therefore high-degree hubs) (Pastor-Satorras et al., 2015). On the contrary, a strong community structure can trap the epidemic in one of the communities, preventing large-scale spreading (Gleeson, 2008; Srinivasan, Dunne, Harte, & Martinez, 2007), which relates to the clinical observation that seizure propagation can often be restricted to one or a few brain lobes (Banerjee et al., 2009), as is the case in focal epilepsy. The fact that epidemic spreading provides a good representation of seizure propagation suggests that other network characteristics that are known to play an important role in epidemic spreading processes, such as temporal changes in connectivity (Masuda & Holme, 2013; Millán, Torres, Johnson, & Marro, 2018; Millán, Torres, & Marro, 2019; Williams, Lacasa, Millán, & Latora, 2022) due to maladaptive plasticity over long time scales (months to years) (De Luca & Papa, 2016), degree correlations (Van Mieghem, Wang, Ge, Tang, & Kuipers, 2010), or dimensionality (Millán, Gori, Battiston, Enss, & Defenu, 2021; Millán, Torres, & Bianconi, 2018; Moretti & Muñoz, 2013), may also affect seizure propagation.

This study is in line with previous works using dynamical models to describe the macroscopic dynamics of seizure propagation. Here, we propose a simple modeling framework based on MEG networks (as opposed to DTI or invasive EEG as previous studies have done (Jirsa et al., 2017; Nissen et al., 2021; Proix et al., 2017; Sip et al., 2021), and in agreement with our previous study (Millán et al., 2022)) and a simple epidemic spreading model to describe the recruitment of new regions into the seizure. Despite the large modeling differences, we were able to replicate the main results of previous works, providing validation for the robustness of the findings. The simplicity of the modeling framework proposed here may be an advantage for its clinical implementation and validation, as well as support future studies looking at the mechanisms underlying seizure dynamics, as it provides an abstract framework that is more mathematically tractable.

Regarding the use of MEG networks as the backbone for seizure propagation, this is based on our previous study (Millán et al., 2022) where we showed that the AEC metric, while based on functional connectivity, retains information on the structural pathways and can be used as a cost-effective proxy for structural connectivity: DTI is not typically part of the standard presurgical evaluation of the patients, has a much higher computational cost than AEC-MEG, and has low sensitivity to long-range connections, in particular interhemispheric ones (Chen et al., 2015). Moreover, AEC-MEG networks capture long-range functional connections that can affect the seizure propagation patterns. Thus, by not correcting for volume conduction the AEC-MEG metric becomes a convenient way to capture both short-range structural and long-range connections.

### Epidemic Spreading Predicts Surgery Outcome

One of the main goals of computational studies of epilepsy surgery is to predict surgery outcome and optimize surgical planning. In our modeling framework, we found that the model, when considering the RA as the epidemic seed, yielded a better fit (as given by the correlation between the modeled and recorded seizures) for SF than for NSF patients, and the difference was significant when considering the population model. Moreover, considering the model fit as a classification parameter led to a good differentiation between the SF and NSF groups, with an AUC of 0.841 for the individual models and 0.886 for the population model, indicating that the goodness of fit could be used as a predictor for surgical outcome. This result also suggests an explanation for the different surgical outcome for the SF and NSF groups as, according to the model, the RA was a better approximation to the SOZ for SF patients, and consequently its removal was more likely to lead to seizure freedom, as was indeed the case. Thus, if a better hypothesis on the SOZ could be made for NSF patients using the computational model, then the resection strategy could also be improved, potentially leading to a better outcome.

It is important to notice, however, that other interpretations are possible. The model fit results are dependent on the SEEG sampling, which may have been inadequate for NSF patients, so that relevant aspects of the seizures were missed (Sip et al., 2021). In this case, the models would not be able to improve the hypothesis on the SOZ, although a poor fitting result could still be used as an indication that more presurgical evaluations are needed, with, for example, alternative spatial sampling. Finally, it is also possible that the worse fit of the model may have been caused by more prevalent nonlinear or multiscale effects for NSF patients, that would make seizure dynamics deviate from a spreading process (Strogatz, 2018). In this case the mismatch would not indicate an error in the SEEG sampling or the surgery planning, but point towards an intrinsic difference in seizure dynamics.

In order to shed light on this question, we made use of the seizure model to generate alternative hypotheses on the SOZ by measuring for each individual ROI the likelihood of generating the observed seizures (Figure 5). At a group level, RA ROIs were significantly more likely to generate the observed seizures than non-RA ROIs, as expected. However, for 8 out of 15 cases RA regions did not show higher seed likelihood than non-RA regions, and the ROI with the maximum seed likelihood did not belong to the RA for any case. This suggests that the most likely seeds according to the model were close to the RA, but did not belong to it. This result is in agreement with other modeling studies that found modeled SOZ that did not completely overlap with the resection areas, even for SF patients (Goodfellow et al., 2016; Proix et al., 2017; Sinha et al., 2017), and is likely associated with the incomplete sampling of the SEEG electrodes. We hypothesize that it may also be related to the finding of pathological hubs whose disconnection from the SOZ can be enough to lead to seizure freedom, even when the SOZ or the pathological hub remain unresected (An et al., 2019; Nissen et al., 2017, 2018; Olmi et al., 2019).

Remarkably, we also found that the difference in goodness of fit between the SF and NSF groups disappeared when considering the optimal single-seed fit. Thus, in the model it is possible to find alternative seed regions for the NSF patients that improve the goodness of fit, and a resection targeting these regions might lead to a better outcome. However, due to the data limitations, and since only four NSF patients were included in this study, we cannot validate this argument.

Finally, we performed a virtual resection analysis to simulate the effect of the resective surgery in silico for each patient (Millán et al., 2022; Nissen et al., 2021; Sinha et al., 2017). We found that virtual resections of the RA led to a significant decrease in seizure propagation. Here we considered the relative decrease in spreading and not seizure extinction to characterize the effect of a resection in the model, as spreading in the model is never null (since the seed is always infected) and the considered virtual resections seldom disconnected the seed completely. Therefore, the relative decrease is more informative than absolute postresection spreading, as it reduces the influence of specific modeling choices. We found that the effect of the resection was predominantly affected by (i) the spreading-to-recovery ratio, (ii) the decrease of betweenness centrality and efficiency of the seed as a consequence of the resection, and (iii) the betweenness centrality and efficiency of the seed *after* the resection. Other properties of the resection and the network also influenced the effect of the resection on spreading, such as the size of the resection area, but their effect was not independent and did not survive the stepwise linear regression analysis. Remarkably, the centrality of the RA has been associated before with surgery outcomes, with the removal of network hubs being associated with seizure freedom (Nissen et al., 2017, 2018, 2021). Here we have found that, more than the centrality of the removed regions, it is the centrality of the SOZ in relation to the RA that will determine the effect of the resection. Given that the gold standard for the actual SOZ in the clinical setting cannot be known, and this often needs to be approximated by the RA, a direct comparison with clinical results is difficult. Prospective studies that include alternative hypothesis for the SOZ, however, can be used to gain more insight in this regard. Finally, other model properties, such as the spreading-to-recovery ratio, also correlated significantly with the relative decrease in spreading, but the effect did not survive the stepwise linear regression analysis, and was mediated by the size of the resection and the centrality of the seed.

In our study, we found that the relative effect of virtual resections was larger for the SF than for the NSF group, and we found AUC values between 0.63 and 0.75 when using the normalized decrease in spreading to classify the patients according to surgical outcome. This again indicates, in agreement with our results based on the goodness of fit, that the RA is a better approximation of the SOZ for SF patients, which is information that can be gained with the model prior to the surgery for a given resection strategy. However, the small sample size in this study limits the predictive power of the results, and the difference in effect between the SF and NSF was not significant. Overall, a larger patient group, including more than one SEEG seizure pattern per patient, would help to improve the predictive power of the model.

### Modeling Considerations and Clinical Application

Epilepsy surgery models need to be individualized for each patient if they are to be of clinical use, in order to take into account the patient-specific brain network and seizure dynamics. This presents a transversal problem in the modeling of epilepsy surgery, as individualizing the models requires extensive data, which is not always available. The existing data on seizure propagation is typically based on SEEG recordings, which, while presenting high temporal and spatial resolution, are limited by sparse spatial sampling, which is known to impact the characterization of the seizures and outcome prediction (Wang et al., 2020) and can lead to bias in the results (Sip et al., 2022). Here we have considered the role of the whole-brain network in seizure propagation by using a whole-brain atlas and MEG data, to reduce the bias due to sparse sampling, but even then only the regions sampled by the SEEG electrodes could be taken into account to fit the model.

In order to simplify the modeling framework, in this study we considered a simple spreading model as the basis for seizure propagation, but there were still specific limitations associated with the modeling scheme. In particular, the propagation of ictal activity captured by the SEEG electrodes is not a binary process, as it was assumed here. On the contrary, ictal activity presents in different qualitative and quantitative forms, and the reduction of the seizure propagation dynamics to a binary activation-inactivation sequence is an oversimplification. Moreover, in order to avoid introducing arbitrary timescales in the model, the seizure patterns only considered the activation order of the ROIs, and not the activation times, which reduces the resolution of the pattern further (as it cannot distinguish fast from slow spreading). We only considered activation times, and not deactivation, since this information was not readily available from the SEEG clinical studies, and deactivation patterns can vary and are not always straightforward to derive. Moreover, considering the deactivation pattern in the current model could be troublesome as (i) the model does not consider time, only “steps” and (ii) we considered a global recovery parameter, so that the average infection time of each infected ROI is the same and variations (in the model) are due to noise. In order to model the deactivation pattern properly, we would thus need to include a realistic timescale and local recovery rates in the model, which would, however, complicate the model definition and increase the dimensionality of the fitting problem, adding to the problem of degeneracy.

Despite the low dimensionality of the model, there was still noise in the fitting method. This noise is intrinsic to the limited clinical data and fitting method, and it is not due to the stochastic nature of the SIR dynamics, which was already taken into consideration: the SIR dynamics were run 10^4^ times per iteration, and 10 iterations were performed, and averaged, for each set of parameters. Moreover, the total correlation metric defined by Equation 6 also takes into account the stochastic nature of the dynamics by weighting every node by its probability of activation in the model. The *parameter noise* can lead to a noisy parameter landscape, with several local maxima. As a consequence, there is the risk of overfitting the individual models, and only limited information can be extracted from the fitting parameters (i.e., from the values of *κ*, *β*, and *γ* leading to the best individual fits).

### Population Model

The population model was defined here as the model (as defined by the set of control parameters) leading to the best average fit over the patient group. Despite its name, this model was still individualized for each patient: it considers the patient-specific network (including the link weights that define the local spreading probabilities) and seed regions. As it is shown in Figure 4, the resulting fit diagram displayed the familiar behavior of a phase transition, with an intermediate region of high correlation (good population fit), separating regions of low correlation (poor population fit). Remarkably, most individual fit points were located in this intermediate region, with the exception of three patients who presented very “bulky” activation patterns (by visual inspection, see the right panel of Figure 2A), that is, in which several ROIs got infected simultaneously. More studies with improved data resolution and larger patients cohorts should be able to establish whether there are actual differences in the dynamical repertoire of these two types of patients.

The average model fit achieved by the population model at its optimal point was much smaller (about one third on average) than the best individual fits. However, the difference between the SF and NSF groups not only still held in this model, but it became stronger and significant ($\bar{C}$(*SF*) − $\bar{C}$(NSF) = 0.267, *p* = 0.02 and AUC = 0.886). This suggests that the loss of detail in the fitting does not affect the main aspects of seizure propagation, and signals toward the possible overfitting of the individual models due to parameter noise. In this case, the population model, even though reducing the overall fit, provided a more reliable description of the system.

A reliable population model would drastically increase the clinical applicability of this framework, as its application would not rely on patient-specific SEEG data. Even though here we have also made use of SEEG data to identify alternative seed regions for the virtual resection analysis, in patients without SEEG recordings the alternative seeds could be based on other modalities of the presurgical evaluation of the patients, that is, the seeds could be based on the suspected irritative zone according to all the presurgical information (including EEG, MRI, MEG) available. SEEG studies are highly invasive, and are avoided in the presurgical evaluation whenever possible. Thus, a computational model that could provide relevant information on surgery outcome without the need to be fitted to patient-specific invasive data would provide a valuable tool. Information from other imaging modalities could potentially also be included, such as ictal EEG recordings of epileptiform abnormalities found in MEG or MRI lesions. This information could be incorporated into the model, for example, as factors that affect the seed likelihood of the involved ROIs.

### Limitations

As we have discussed above, modeling of seizures presents inherent limitations associated with the choices of the dynamical model and fitting procedures (Hashemi et al., 2020; Jha, Hashemi, Vattikonda, Wang, & Jirsa, 2022; Sip et al., 2022). In our case, this translated into difficulties defining alternative seeds and characterizing virtual resections. Identifying seed regions dramatically increases the dimensionality of the fitting problem, even when considering linear approximations as we have done here. Consequently, we decided to split the optimization process in two and fit the model parameters with a fixed seed (the resection area), and then find optimal seed regions with these global model parameters. Although this could lead to suboptimal results for the individual seed analysis, and may influence our findings (such as the lack of overlap between the optimal seeds and the resection areas), it provides a more robust analysis method. Moreover, it would not be realistic to assume different global regimes depending on the seed region either. However, these simplifications may have affected the seed-likelihood maps, as we found, for instance, the most likely seed did not belong to the resection area for any patient. Estimating seed-probability maps for seeds of increasing sizes becomes a combinatorial problem that soon loses tractability. The use of optimization algorithms (such as simulated annealing, genetic algorithms, or deep learning, for instance (Millán et al., 2022; Nissen et al., 2021)) would reduce the computational burden but still be affected by an exponentially larger number of local maxima as the seed size increases, due to the parameter noise. In our analysis we have opted for a linearization approach, characterizing the effect of single seeds and following a recursive method to derive seeds of increasing sizes. Deriving more detailed seeds would require larger amounts data, for instance, by considering several seizures per patient. The degeneracy can also be a problem for identifying optimal resection strategies. To minimize this issue in the model, one could, for instance, consider only viable resection strategies, for example, avoiding eloquent cortex or nonfocal resections. In this case, however, the degeneracy could be advantageous for surgical planning, if different resection strategies could be found, since this would allow the surgeon to consider the one least likely to cause severe side effects.

When defining the network backbone, we have decided to exclude strong negative connections (before normalization, which would translate into normalized weigth values close to 0) from the network through the thresholding procedure. It was designed in this manner in analogy to previous studies (Millán et al., 2022) and to allow for a more straightforward comparison with structural networks. In fact, we do not consider here a pure functional connectivity metric, as the AEC is strongly influenced by volume conduction. Consequently, negative associations are affected by volume conduction and difficult to interpret. It is also unclear whether negative correlations facilitate seizure propagation. It would be an interesting point for future studies to consider independently the role of structural, positive functional, and negative functional connections in seizure propagation. However, we do not expect the main results from this study to change significantly by the inclusion of negative correlations. As we showed in our previous study (Millán et al., 2022), where we compared the model fit with patient-specific and population networks, the main results held and in fact were not significantly distinguishable, a result that has also been found by other groups in different settings (Proix et al., 2017).

The use of SEEG data to fit the model posses another limitation for its clinical use, given that SEEG recordings are highly invasive and not always part of the presurgical evaluation. A reliable population model (that can be individualized for each patient by considering their individual brain connectivity) would allow us to also use the model for patients without SEEG recordings, as we have discussed above. Moreover, as we have shown in this study, such a model can also help in reducing parameter noise, leading to more robust results than the individual models.

Another main limitation of the study is the small size of the patient group, which complicates the validation of the results. For instance, the virtual resection analysis points toward a larger decrease in seizure propagation after the virtual resections for the SF group, but the difference is not significant. In this study we increased the patient cohort from 10 patients in Millán et al. (2022) to 15 (50% increase), but the size of the NSF group only increased by one case, which is still insufficient for an accurate statistical analysis. A larger cohort would allow us to improve the classification analysis to clarify our findings. Moreover, increasing the patient group size would also improve the formulation of the population model.

Modeling of virtual resections suffers from some inherent limitations as well. Model degeneracy is also a problem to verify the model findings, as different situations can lead to the same observable effects, and, in fact, one can only know whether the performed surgery was successful or not, but not what were the underlying causes leading to either scenario. The simulation of the resection also posses modeling limitations. Virtual resections are typically modeled by removing or disconnecting nodes or links from the network (Banerjee et al., 2009; Jirsa et al., 2017; Kini et al., 2019). However, this does not account for the generalized effect that a local resection can have on the network (Demuru et al., 2020) nor does it consider plasticity mechanisms (Holtmaat & Svoboda, 2009; Millán, Torres, Johnson, & Marro, 2018, 2021; Stam, 2014; Stam, Hillebrand, Wang, & Van Mieghem, 2010), which are known to occur following brain lesions and resections (Sidhu et al., 2016; Tao & Rapp, 2021). Virtual resection analyses also suffer from degeneracy, as different resections might have the same effect on the model. An even more fundamental limitation is the difficulty of the validation of the results, as different resection strategies cannot be tested clinically. Validation must always be done indirectly, by comparing the model predictions (regarding, for instance, the location of the SOZ or surgery outcome) with the clinical results (Goodfellow et al., 2016; Lopes et al., 2017; Millán et al., 2022; Nissen et al., 2021; Sinha et al., 2017). In this work, we have made use of multimodal patient-specific data to optimize and validate the model and, as a final validation mechanism, we have considered surgery outcome. This can only be the first step, however, as the ultimate goal is to use the computational models to aid epilepsy surgery planning. Prospective or pseudoprospective studies in which the models are used before or without knowledge of the surgery to predict outcome at an individual level (i.e., not only at a group level) will be necessary in the future to test the applicability of the model on a clinical setting.

### Conclusion and Outlook

Epidemic spreading models fitted with patient-specific data reproduce the individual seizure propagation patterns. This simple framework is sufficient to encode the fundamental aspects of seizure propagation on brain networks. Our results highlight that such individualized computational models may aid epilepsy surgery planning by identifying alternative seed regions and/or resection strategies, with the ultimate goal of improving surgery outcome rates.

## METHODS

### Patient Group

We retrospectively analyzed 15 patients (9 females) with refractory epilepsy. All patients had undergone resective surgery for epilepsy at the Amsterdam University Medical Center, location VUmc, between 2016 and 2019. All patients had received a magnetoencephalography (MEG) recording, had undergone an SEEG (stereo-electroencephalography) study, including postimplantation CT scans, and underwent pre- and postsurgical magnetic resonance imaging (MRI). All patients gave written informed consent and the study was performed in accordance with the Declaration of Helsinki and approved by the VUmc Medical Ethics Committee.

The patient group was heterogeneous with temporal and extratemporal resection locations and different etiology (see Table 2 for details). Surgical outcome was classified according to the Engel classification at least 1 year after the operation (Engel, Van Ness, Rasmussen, & Ojemann, 1993). Patients with Engel class 1A were labeled as seizure free (SF), and patients with any other class were labeled as nonseizure free (NSF). Four patients were deemed NSF.

Part of this patient cohort was also included in our previous study modeling seizure propagation and epilepsy surgery (Millán et al., 2022). For these patients we used the data derived in the previous study (e.g., AEC-MEG network, SEEG seizure pattern, resection area, surgical outcome), but all analyses were performed independently from the previous study.

### Individualized Brain Networks

The individualized computer model was based on the brain network of each patient, which was reconstructed in the Brainnetome Atlas (BNA) (Fan et al., 2016) from MEG scans. The use of MEG-derived networks as the backbone for seizure propagation was motivated in our previous study (Millán et al., 2022), where we showed that they allowed for a good reproduction of clinically recorded seizure-propagation patterns. The networks include deep regions such as the hippocampus, which is important to adequately capture the propagation of temporal lobe seizures, for instance. Previous studies have validated the the sensitivity of MEG that MEG is sensitive to hippocampal activity (Engels et al., 2016; Hillebrand, Nissen, et al., 2016; Meyer et al., 2017; Pizzo et al., 2019; Ruzich, Crespo-García, Dalal, & Schneiderman, 2019; Tierney et al., 2021) and can be used to reconstruct the activity of subcortical structures (Andersen, Jerbi, & Dalal, 2020; Krishnaswamy et al., 2017) albeit with varying spatial resolution (from 1–20 mm) (Barnes, Hillebrand, Fawcett, & Singh, 2004; Hillebrand & Barnes, 2005). In this study we considered a well-established cortical and subcortical atlas, the Brainnetome (Fan et al., 2016), that has been well validated for MEG (Joshi & Zaveri, 2022; Pourmotabbed, Wheless, & Babajani-Feremi, 2020; Yuan et al., 2019). The network processing framework was exactly the same as in our previous study (Millán et al., 2022), in particular:

▪  **Preoperative MRI scans** were used for coregistration with the MEG data. MRI T1 scans were acquired on a 3T whole-body MR scanner (Discovery MR750, GE Healthcare, Milwaukee, Wisconsin, USA) using an eight-channel phased-array head coil. Anatomical 3D T1-weighted images were obtained with a fast spoiled gradient-recalled echo sequence. During reconstruction, images were interpolated to 1 mm isotropic resolution.
▪  **MEG recordings** were obtained during routine clinical practice using a whole-head MEG system (Elekta Neuromag Oy, Helsinki, Finalnd) with 306 channels consisting on 102 magnetometers and 204 gradiometers. The patients were in supine position inside a magnetically shielded room (Vacummshmelze GmbH, Hanau, Germany). Typically, three eyes-closed resting-state recordings of 10 to 15 minutes each were acquired and used in the presurgical evaluation for the identification and localization of interictal epileptiform activity. The first of these recordings of sufficient quality was used here to generate the brain network. The data were sampled at 1250 Hz, and filtered with an anti-aliasing filter at 410 Hz and a high-pass filter of 0.1 Hz. The head’s position relative to the MEG sensors was determined using the signals from four or five head-localization coils that were recorded continuously. The positions of the head-localization coils and the outline of the scalp (roughly 500 points) were measured with a 3D digitizer (Fastrak, Polhemus, Colchester, VT, USA).
▪  **MEG preprocessing**. The temporal extension of signal space separation (tSSS) (Taulu & Hari, 2009; Taulu & Simola, 2006) was used to remove artifacts using Maxfilter software (Elekta Neuromag, Oy; version 2.1). For a detailed description and parameter settings see Hillebrand, Fazio, de Munck, and van Dijk (2013). The MEG data were filtered in the broadband (0.5–48.0 Hz).
▪  **MEG and MRI coregistration**. The points on the scalp surface were used for coregistration of the MEG scans with the anatomical MRI of the patient through surface-matching software. A single sphere was fitted to the outline of the scalp and used as a volume conductor model for the beamforming approach.
▪  **Source reconstruction: beamforming**. Neuronal activity was reconstructed using an atlas-based beamforming approach, modified from Hillebrand, Barnes, Bosboom, Berendse, and Stam (2012), to reconstruct the time series of neuronal activation of the ROI centroids (Hillebrand, Tewarie, et al., 2016). We considered the 246 ROIs of the BNA atlas (Fan et al., 2016), whose centroids were inversely transformed to the coregistered MRI of the patient. Then, a scalar beamformer (Elekta Neuromag Oy; beamformer; version 2.2.10) was applied to reconstruct each centroid’s time series, as detailed elsewhere (Hillebrand, Tewarie, et al., 2016).
▪  **Processing**. The time series of each centroid were visually inspected for epileptiform activity and artifacts. On average, 58 ± 11 interictal and artefact-free epochs of 16,384 samples were selected for each patient. The epochs were further analyzed in Brainwave (version 0.9.151.5) and were downsampled to 312 Hz and filtered in the broadband (0.5–48 Hz).
▪  **Functional networks** were generated considering each brain region as a node. The elements *w*_*ij*_ of the connectivity matrix, indicating the strength of the connection between ROIs *i* and *j*, were estimated by the AEC (Brookes et al., 2011; Bruns, Eckhorn, Jokeit, & Ebner, 2000; Colclough et al., 2016; Hipp, Hawellek, Corbetta, Siegel, & Engel, 2012). The uncorrected AEC (i.e., without correcting for volume conduction) connectivity metric was selected as it maintains information on the structural connectivity pattern, while, by not correcting for volume conduction, it maintains information on the structural connections, which are mainly determined by the distance between each ROI pair. In fact, in Millán et al. (2022) we validated the relationship between AEC-MEG and structural networks by comparing them with a well validated model for structural connectivity: the exponential distance rule (EDR) network. Based on animal studies, the EDR specifies that the weights of structural connections in the brain, *w*_*ij*_, decay exponentially with the distance between ROIs *d*_*ij*_ (Ercsey-Ravasz et al., 2013; Gămănuţ et al., 2018; Theodoni et al., 2022), that is, *w*_*ij*_ ∝ *exp*(−*αd*_*ij*_). Recent studies have repeatedly found that the EDR reproduces human DTI data well (Deco & Kringelbach, 2020; Deco et al., 2021; Roberts, Perry, Roberts, Mitchell, & Breakspear, 2017). In Millán et al. (2022) we compared AEC-MEG and EDR networks for a 10 patient cohort (which is part of the patient cohort in the current study) by calculating the Pearson’s correlation coefficient between the matrix entries (see Supplementary Figure S1 of Millán et al., 2022) and found a strong correlation (*R*^2^ = 0.50) between the two for a decay exponent *α* = 0.052. Moreover, AEC-MEG networks also include long-range structural connections that can aid seizure propagation. Thus, it is a convenient way to combine both effects in one network. AEC values were rescaled between 0 (perfect anticorrelation) and 1 (perfect correlation), with 0.5 indicating no coupling (Briels et al., 2020). Functional networks were thresholded at different levels *θ* indicating the percentage of remaining links in the network, and the resulting average connectivity *κ* of the network, indicating the average number of links that each node had, was determined. Notice that the networks were thresholded but not binearized, so that *w*_*ij*_ takes values between 0 and 1. An exemplary adjacency matrix is shown in Figure 1 under “Brain Network.”

### Individualized Propagation Pattern

All patients underwent stereo-electroencephalography (SEEG) electrode implantation. The number and location of the intracerebral electrodes (Ad-Tech, Medical Instrument Corporation, USA; 10–15 contacts, 1.12-mm electrode diameter, 5-mm intercontact spacing; and DIXIE, 10–19 contacts, 0.8-mm electrode diameter, 2-mm contact length, 1.5 mm insulator length, 16–80.5 mm insulator spacer length) were planned individually for each patient by the clinical team, based on the location of the hypothesized SOZ and seizure propagation pattern. The number of electrodes per patient (see Table 1 for details) varied between 9 and 15 (average = 12.1 ± 1.8) and the total number of contacts between 194 and 99 (average = 121 ± 24).

The preprocessing and analysis of the SEEG data is analogous to that in Millán et al. (2022). The locations of the SEEG contact points were obtained for each patient from the postimplantation CT scan (containing the SEEG electrodes) that was coregistered to the preoperative MRI scan using FSL FLIRT (version 4.1.6) 12 parameter afine transformation. Each electrode contact point was assigned the location of the nearest ROI centroid. Because BNA ROIs are in general larger than the separation between contact points, different contact points can have the same ROI assigned.

The activation time of each sampled ROI was determined according to the SEEG recording as follows. First, the onset time of ictal activity was identified for each SEEG channel by a clinician expert, and the contact points were ordered according to their activation time into activation steps. When two or more contact points became active at the same time, they were assigned the same activation step. In this manner a seizure pattern was built from one typical seizure for each patient. This activation pattern was then translated into the BNA space, so that the each sampled ROI *i* was assigned an activation step corresponding to the smallest activation step of the contact points within the ROI, in agreement with previous studies (Millán et al., 2022). This constituted the SEEG seizure pattern.

### Seizure Propagation Model

#### SIR dynamics.

Seizure propagation was modeled using the Susceptible-Infected-Recovered (SIR) model (Pastor-Satorras et al., 2015) following our previous study in Nissen et al. (2021). In that study the model parameters were set ad hoc for each patient so as to set the system in the supercritical regime (ensuring that 98% of the simulated seizures would propagate over the whole network). Here, we made use of SEEG ictal recordings to fit the SIR model parameters to the patient-specific propagation patterns, following a similar procedure to our previous work using the simple Susceptible-Infected model (Millán et al., 2022).

Simulation of the epidemic spreading process on the network took place as follows. Initially, all nodes were set in the susceptible state *S*, except for a set of *seed* nodes in the infected state *I*. At each subsequent step, each infected node could propagate the infection to any of its neighbours with probability *βw*_*ij*_, where *β* characterizes the global spreading rate and *w*_*ij*_ the link weight as defined above. Each infected node had a probability *γ* of recovering to the *R* state. Referring to the state of node *i* at time *t* as *X*_*i*_(*t*), where *X* can take values *S*, *I*, *R*, the probability that node *i* is in each state at time *t* > 1 is given by (Barrat et al., 2008; Newman, 2002; Pastor-Satorras et al., 2015):

$$P\left(X_{i}\left(t\right)=S\right)=\left[1-p_{i}\left(S\to I,t\right)\right]P\left(X_{i}\left(t-1\right)=S\right),$$ (1)

$$P\left(X_{i}\left(t\right)=I\right)=\left(1-\gamma \right)P\left(X_{i}\left(t-1\right)=I\right)+p_{i}\left(S\to I,t\right)P\left(X_{i}\left(t-1\right)=S\right),$$ (2)

$$P\left(X_{i}\left(t\right)=R\right)=P\left(X_{i}\left(t-1\right)=R\right)+\gamma P\left(X_{i}\left(t-1\right)=I\right),$$ (3)

where

$$p_{i}\left(S\to I,t\right)=\beta \sum_{j}w_{ij}P\left(X_{j}\left(t-1\right)=I\right)+\sum_{n=2}^{k_{i}}{\left(-1\right)}^{n+1}\sum_{\begin{array}{c} j_{1},\ldots ,j_{n}\\ j_{n}<j_{n-1}<\ldots <j_{1} \end{array}}\beta^{n}\prod_{k}^{n}w_{ik}$$ (4)

is the probability that a susceptible node *i* becomes infected by any of its neighbors. The first term corresponds to the probability that any of the neighbours of *i* transmit to it the infected state, whereas the second term takes into account that *i* can only be infected once.

Depending on the network structure, the epidemics can show different spatiotemporal spreading profiles described by the probability *p*_*i*_(*t*) that each ROI *i* becomes infected at step *t*. SIR dynamics were simulated in custom-made MATLAB algorithms using Monte-Carlo methods, with *N*_*R*_ = 10^4^ iterations of the algorithm for each configuration to assure convergence.

#### Individualized propagation model.

The seizure propagation model was fitted for each patient by comparing the spatiotemporal propagation pattern in the model to the patient’s clinical seizure pattern (constructed as described above), when the RA was used as the seed for epidemic spreading. We followed a similar methodology as in our previous study (Millán et al., 2022) but adapted to the SIR model, instead of the simple Susceptible-Infected. Thus, the results of the two fit methods are not directly comparable. The mean activation time for each ROI was calculated as *t*_*i*_ = $\sum_{t=0}^{T}$ *p*_*i*_(*t*), where *T* is the maximum integration time; *t*_*i*_ described the activation sequence of the ROIs during a modeled seizure. By sorting the ROIs according to their mean activation time, we defined the SIR propagation pattern, which indicated the activation order of the involved ROIs. Given that not all BNA ROIs were sampled by the SEEG electrodes, *t*_*i*_ was subsampled to the sampled ROIs. This constituted the SIR seizure pattern, as shown in Figure 1 (under “SIR Seizure Pattern”), which could be compared with the SEEG seizure pattern.

The goodness of fit of the model was estimated by taking into account two factors, as illustrated in Figure 2. The first one is the correlation between activation orders of ROIs that became infected both in the SEEG and SIR patterns (see Figure 2A). In order to take into account the noisy nature of the SIR dynamics, such that not the same ROIs get infected in each realization, we considered the weighted Pearson’s correlation coefficient, *C*_*w*_. As correlation weights we used the fraction of realizations that each ROI *i* got infected during a modeled seizure, *P*_*IR*_(*i*). Thus, ROIs that were consistently involved in the spreading weighted more in the correlation than ROIs that were only rarely involved.

The second factor *P*_overlap_ (see Figure 2B) computed the overlap between active and inactive ROIs in the two patterns, to also take into account the actual extension of the seizures, that is,

$$P_{\text{overlap}}=N_{\text{SEEG}}^{-1}\left[\sum_{i\in 𝕊}P_{\text{IR}}\left(i\right)+\sum_{i\in ℍ}\left(1-P_{\text{IR}}\left(i\right)\right)\right]=P_{\text{act}}+P_{\text{inact}},$$ (5)

where *N*_SEEG_ is the number of ROIs sampled by the SEEG electrodes (on average = 43.6 ± 7.9), and 𝕊 and ℍ are, respectively, the sets of active (in the seizure state) and inactive (in the healthy state) ROIs in the SEEG pattern. Thus, the total correlation between the two patterns was defined as

$$C=C_{w}\cdot P_{\text{overlap}},$$ (6)

This metric equals 1 in case of exactly equal activation patterns, 0 in the case of null overlap or correlation, and −1 in the case of complete anticorrelation of activation times (but equal seizure areas). We note, however, that *C* decays from 1 faster than a simple correlation metric when there are discrepancies between the patterns, since it takes into consideration not only the activation times but also the activation areas.

In order to fit the model to the SEEG data, the RA was set as the seed of the epidemics, and the model parameters (*κ*, *β*, *γ*) were fitted to the data by maximizing *C*, independently for each patient. In order to do so, the SIR dynamics was simulated for a range of values of the free parameters (*β*, *γ* ∈ {10^−4^, 10^−3^, 10^−2^, 10^−1^}, *κ*/*N* = {0.025, 0.05, 0.10, 0.20, 0.30}), leading to the three-dimensional fit-map *C* (*κ*, *β*, *γ*). Two exemplary fit-maps are shown in Supporting Information Figure S1. In order to minimize noise effects and be able to estimate the error in the measure, *C* values were averaged over 10 iterations of the model (each comprising 10^4^ repetitions for the SIR dynamics), and the error in the measure was defined as the standard deviation across these 10 iterations. We selected a large value of *T* compared to the network size (*T* = 1,000), which was enough to guarantee that the majority of the simulations reached the stationary state before the simulation was ended.

#### Population model.

We defined the population model as the model that provided the best fit overall, by averaging the fit results of all patients. We first estimated the algebraic average population fit for each parameter configuration: $\bar{C}$(*β*, *γ*, *κ*) = $N_{\text{pats}}^{\left(-1\right)}$ ∑_*pat*_ *C*_*pat*_(*β*, *γ*, *κ*). Then, we identified the parameter set (β*, γ*, κ*) leading to the maximum value of $\bar{C}$. This pair of values defined the population model. All patient fits were weighted the same for the average to avoid overfitting or biasing the fit to one particular population. However, since we performed the algebraic average of individual fit values, patients with better fits had a larger impact in the values of β*, γ*, and κ*. The local spreading rates were defined for each patient based on their individual AEC-MEG network, and the seed regions were based on the patient-specific resection area.

#### Alternative seed regions.

Alternative seed regions were found with the model by considering each ROI *R* as the single seed of epidemic spreading, once the model was fitted to the patient-specific seizure propagation patterns (i.e., we used the global model parameters derived from the model fit using the resection area as the seed when searching for alternative seed regions). Then, the seed likelihood of the ROI was defined as the total correlation between the SEEG and SIR patterns, *C*_*R*_. Only ROIs leading to spreading were included in the analyses. From this analysis we estimated the best fit given by a single ROI, referred to as “*Best*” and the average value of the fit given by the RA regions, when considered individually as seeds, referred to as 〈*RA*〉. Finally, for comparison purposes we also estimated the average model fit given by random seeds (*N* = 20) of the same size of the RA, referred to as *RND*.

### Simulation of Resections

We conducted virtual resections of the ROIs that were part of the RA to simulate the effect of the surgery in the model. In order to do this, the nodes belonging to the RA were disconnected from the network by setting to 0 all their connections. The effect of each resection was characterized by the normalized decrease in spreading in the resected network (*IR*_*R*_) with respect to the original (*IR*_0_) spreading: *δ*_*R*_ = (*IR*_0_ − *IR*_*R*_)/*IR*_0_, where *IR* is the fraction of nodes that became infected at any point during the modeled seizure, that is, *IR* = *I*(*t* → ∞) + *R*(*t* → ∞).

The model parameters were chosen as the optimal fitting parameters for each patient, whereas the seed regions were defined following a recursive optimization method. Starting from the best single seed, all possible combinations of this node with the remaining 245 nodes were tested as spreading seeds, and the one leading to the best model fit (i.e., maximum total correlation) was chosen. This process was subsequently iterated until seeds of size 5 were obtained. To account for the large differences in seed sizes and connectivity, we rescaled the spreading rate by the fraction between the out-connectivity (number of links to the rest of the network) of the RA and the considered seed.

To understand what network and model characteristics relate to the effect of the resection on spreading, we estimated the Pearson correlation coefficient between the normalized decrease in spreading due to the resection, *δ*_*R*_, and different network, and model metrics. In particular, as model metric we considered the spreading-to-recovery ratio, *βκ*/*γ*, which takes into account that spreading is enhanced by *β* and *κ* and slowed by *γ*. As network metrics we considered the properties of resection area, seed regions and whole network. In particular, we considered the size *S*_*X*_ (number of nodes of the node set *X*), out-connectivity *E*_*X*_ (number of links from *X* to the rest of the network), average betweennees centrality BC_*X*_ (number of short paths that cross through *X*), average clustering coefficient *c*_*X*_ (fraction of closed triangles), and average efficiency *F*_*X*_ (network-based distance from *X* to the rest of the network nodes), where *X* is the considered node set (i.e., either the RA, the seed or the whole network). For the whole network we only considered the average clustering and efficiency as the other metrics are fixed. For the seed we considered three scenarios: the baseline level (0, prior to the resection), the postresection level (*R*), and the decrease due to the resection, Δ.

In order to identify the most relevant model variables to predict the effect of a virtual resection in the model, we performed a stepwise linear regression model analysis. We used the *Statistical and Machine Learning Toolbox* with standard settings (*stepwiselm* function with default settings). Only linear effects, and no interaction effects, were allowed in the linear model. As dependent variable, we considered the normalized effect of the resection in logarithmic scale, log(*δ*_*IR*_). As independent variables, we considered the same network and model metrics as in the pairwise correlation analyses.

### Statistics

The weighted correlation coefficient was used to determine the correlation between the SEEG and SIR seizure propagation patterns. For comparisons between resected and nonresection areas, and between different seed definitions, we used paired Student’s *t* tests, whereas for comparisons between SF and NSF patients, we used unpaired Student’s *t* tests. Significance thresholds for statistical comparisons were set at *p* < 0.05.

We performed a receiver-operating characteristic (ROC) curve analysis to study the patient classification based on the goodness of fit of the models and the normalized effect of virtual resections. A positive result was defined as good (SF) outcome.

In order to account for the noise in the SIR model, the dynamics were run 10^4^ to attain each SIR seizure pattern, and this was repeated 10 times to obtain averaged values. The errors were defined as the standard deviation between the 10 realizations of the model. The same procedure was used in the virtual resection analysis.

### Data Availability

The data used for this manuscript are not publicly available because the patients did not consent for the sharing of their clinically obtained data. Requests to access to the datasets should be directed to the corresponding author. All user-developed codes are publicly available in https://github.com/anapmillan/computer_model_for_epilepsy.

## SUPPORTING INFORMATION

Supporting information for this article is available at https://doi.org/10.1162/netn_a_00305.

## AUTHOR CONTRIBUTIONS

Ana P. Millán: Conceptualization; Data curation; Formal analysis; Investigation; Methodology; Project administration; Software; Validation; Visualization; Writing – original draft; Writing – review & editing. Elisabeth C. W. van Straaten: Conceptualization; Data curation; Funding acquisition; Investigation; Methodology; Project administration; Resources; Supervision; Validation; Writing – review & editing. Cornelis J. Stam: Conceptualization; Funding acquisition; Investigation; Methodology; Project administration; Resources; Software; Supervision; Writing – review & editing. Ida A. Nissen: Conceptualization; Funding acquisition; Investigation; Software; Writing – review & editing. Sander Idema: Conceptualization; Data curation; Funding acquisition; Investigation; Writing – review & editing. Johannes C. Baayen: Conceptualization; Funding acquisition; Resources; Writing – review & editing. Piet Van Mieghem: Conceptualization; Funding acquisition; Writing – review & editing. Arjan Hillebrand: Conceptualization; Funding acquisition; Investigation; Methodology; Project administration; Resources; Software; Supervision; Visualization; Writing – review & editing.

## FUNDING INFORMATION

Ana P. Millán, ZonMW (https://dx.doi.org/10.13039/501100001826), Award ID: 95105006. Ida A. Nissen, ZonMW (https://dx.doi.org/10.13039/501100001826), Award ID: 95105006. Ana P. Millán, Epilepsy Foundation (NL) , Award ID: 95105006. Ida A. Nissen, Epilepsy Foundation (NL), Award ID: 95105006.

## TECHNICAL TERMS

Epileptogenic zone: Region(s) in the brain that needs to be removed to stop the occurrence of epileptic seizures.
Virtual resection: Simulation of a resection strategy in a computational model by removing or disconnecting the regions included in the resection.
Seizure onset zone: Brain region (or regions) from which seizures are generated.
MEG: Magnetoencephalography, noninvasive functional recording that measures the magnetic field generated by brain activity.
SEEG: Stereo-tactical electroencephalography, consisting of the recording of local brain activity through the generated electrical fields via depth electrodes that placed inside the brain.
Susceptible-Infected-Recovered model: Epidemiological model that describes the spreading of an agent over a network, accounting for the infection and recovery processes.
Resection area: Set of brain regions removed during epilepsy surgery.
