# Supplementary material for: The role of epidemic spreading in seizure dynamics and epilepsy surgery
Source: Netw Neurosci. 2023 Jun 30;7(2):811–43. doi: 10.1162/netn_a_00305 (PMC10312291; doi:10.1162/netn_a_00305)
Supplement: Supplementary file 1 [file netn-7-2-811-s001.pdf]

Millan, A. P., van Straaten, E. C. W., Stam, C. J., Nissen, I. A., Idema, S., Baayen, J. C., Van Mieghem, P. & Hillebrand, A. (2023). Supporting information for "The role of epidemic spreading in seizure dynamics and epilepsy surgery." *Network Neuroscience*. Advance publication. https://doi.org/10.1162/netn_a_00305

Supplementary Information to: "The role of epidemic spreading in seizure dynamics and epilepsy surgery"

Ana. P. Millán[1,*], Elisabeth C.W. van Straaten[1,4,5], Cornelis J. Stam[1,3,5], Ida A. Nissen[1], Sander Idema[2,4,6], Johannes C. Baayen[2,4,6,7], Piet Van Mieghem[8], and Arjan Hillebrand[1,3,4]

[1]Amsterdam UMC location Vrije Universiteit Amsterdam, Department of Clinical Neurophysiology and MEG Center, De Boelelaan 1117, Amsterdam, The Netherlands
[2]Amsterdam UMC, Vrije Universiteit Amsterdam, Department of Neurosurgery, De Boelelaan 1117, Amsterdam, The Netherlands
[3]Amsterdam Neuroscience, Brain Imaging, Amsterdam, The Netherlands
[4]Amsterdam Neuroscience, Systems & Network Neurosciences, Amsterdam, The Netherlands
[5]Amsterdam Neuroscience, Neurodegeneration, Amsterdam, The Netherlands
[6]Amsterdam Neuroscience, Cancer Biology and Immonology, Amsterdam, The Netherlands
[7]Amsterdam Neuroscience, Imaging and Biomarkers, Amsterdam, The Netherlands
[8]Faculty of Electrical Engineering, Mathematics and Computer Science, Delft University of Technology, Delft, The Netherlands
[*]Corresponding author: a.p.millanvidal@amsterdamumc.nl

1 Model fitting

Figure S1 shows the fit results for two exemplary cases, as indicated in the caption. Best fit parameters (used during the analysis) are indicated by solid circles.

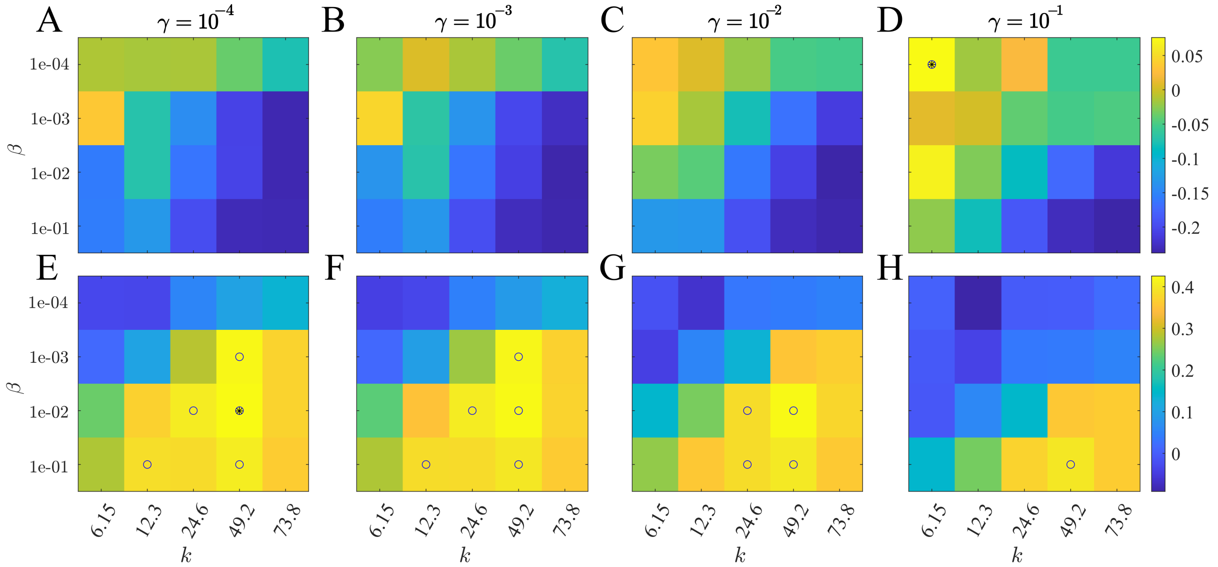

Figure S1: Model fit. Fit results for two exemplary patients (cases 10 and 15, respectively on the top and bottom rows) as given by $C(\beta, \kappa)$ for different values of γ. The full circle marker indicates the best fit, and empty markers indicate fit points with at least 90% of the maximum value.

2 Population model

For the definition of the population model, we measured the average fit across the population for each set of parameter values, $\bar{C}(\beta, \gamma, \kappa)$. The resulting fit diagrams are shown in figure S2 for the whole (top row), seizure-free (mid row) and non-seizure-free (bottom row) groups. In order to aid visualization, in figure S3 we show the same fit diagrams in a 2D representation by combining β and κ into a single parameter, the re-scaled spreading rate $\beta\kappa$, quantifying the effective global spreading rate. In this figure we also show the standard deviation and signal-to-noise ratio to illustrate the variation across the population.

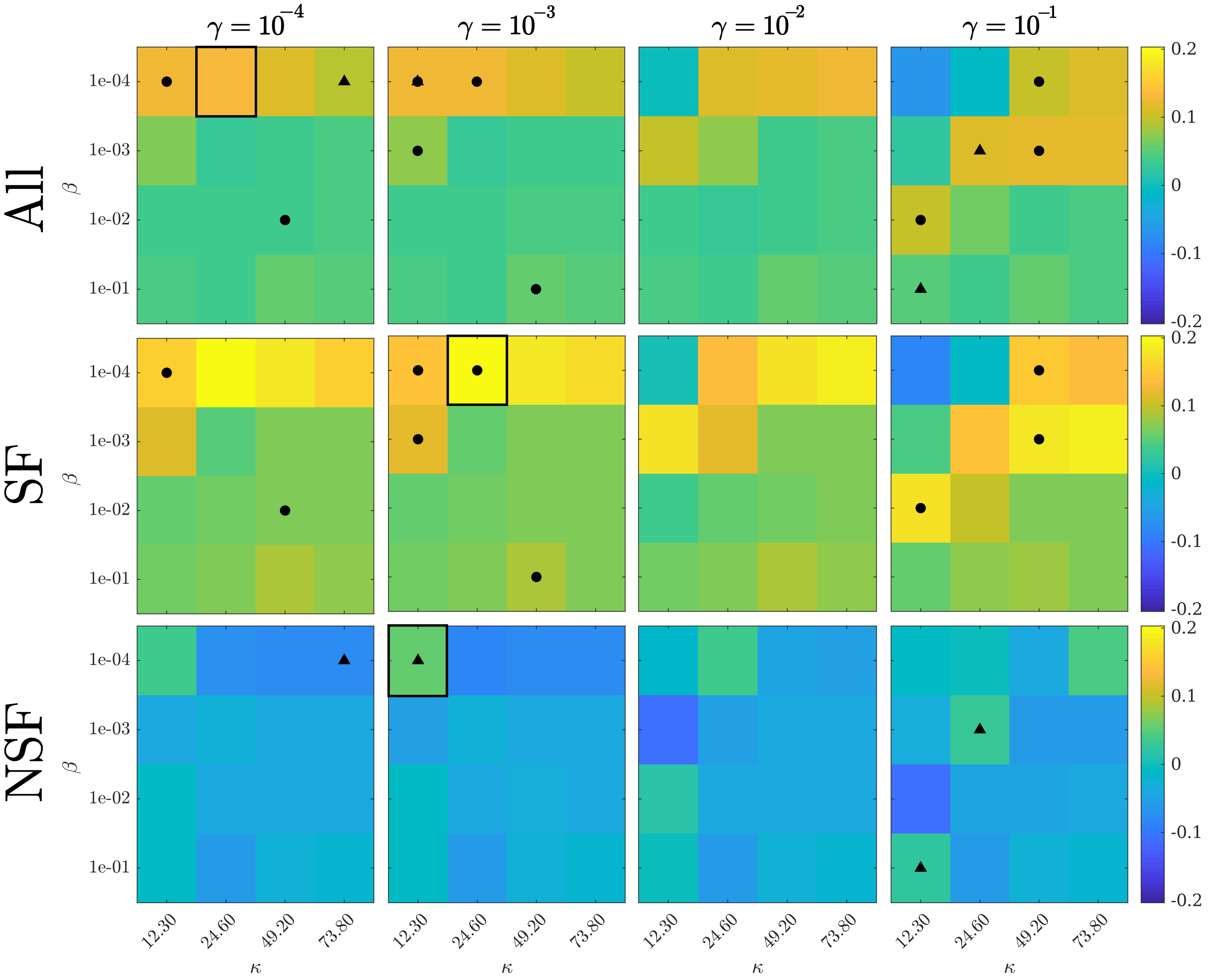

Figure S2: Population model: $3D$ representation. Here we show the average fit maps for the whole (top, "All"), SF (middle) and NSF (bottom) groups, in terms of the average model fit for each set of parameter values (γ, β, κ). Each panel corresponds to a fixed value of γ. For each group we also show the individual best fit with circles and triangles corresponding respectively for SF and NSF patients. The black boxes mark the best fit (i.e. highest goodness-of-fit).

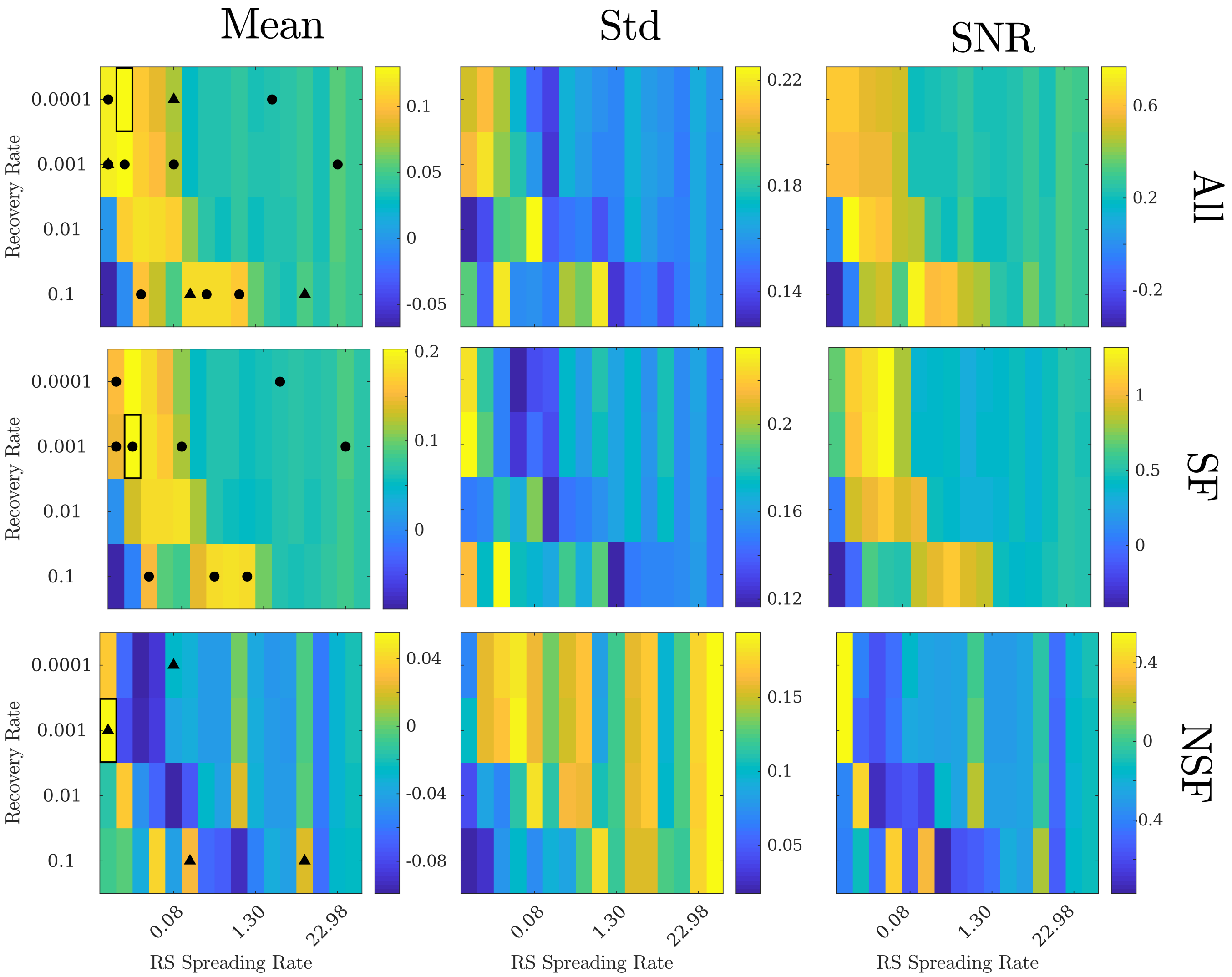

Figure S3: Population model. Here we show the average fit maps for the whole (top, "All"), SF (middle) and NSF (bottom) groups, in terms of the average model fit (left, "Mean"), its standard deviation (center, "Std") and the signal to noise ratio (right, "SNR"), given by the ratio between the average values and the standard deviation. The axis indicate the recovery rate γ and the re-scaled spreading rate, $\beta\kappa$
. For each group we show the individual best fit in the left panels with circles and triangles respectively for SF and NSF patients. The black boxes mark the best fit (i.e. highest goodness-of-fit). The data shown in the left column is exactly the same as that of figure S2 but combining β and κ in one parameter.

3 Alternative seizure onset zones

3.1 Comparison with the resection areas

In this section we report on the details of the statistical analyses in section 2.4: "Alternative Seizure Onset Zones" of the main text. Table S1 corresponds to the comparison between the seed-likelihood of resected (RA) and non-resected (NRA) areas. All results correspond to un-paired t-tests. In table S2 we report the results from the statistical comparisons between different seed definitions.

Case	C_{RA}	C_{NRA}	Diff.	t	p	df	Sig.
1	0.300	0.036	0.261	1.744	0.08	89	*
2	0.173	−0.018	0.191	2.73	0.07	186	*
3	−0.205	0.0186	−0.223	−1.10	0.22	27	
4	−0.005	−0.001	−0.003	−0.32	0.75	193	
5	−0.005	0.039	−0.044	−0.81	0.42	162	
**6**	0.067	0.029	0.038	1.48	0.14	239	
7	0.077	−0.008	0.085	1.76	0.08	242	*
8	0.0233	−0.007	0.030	0.59	0.56	161	
9	0.010	−0.198	0.208	5.56	$< 10^{-4}$	244	**
**10**	−0.016	−0.003	−0.013	−0.41	0.69	58	
**11**	0.173	−0.038	0.211	4.01	0.001	244	**
12	0.340	0.100	0.240	0.63	0.54	10	
13	0.050	−0.111	0.161	8.92	$< 10^{-4}$	244	**
**14**	0.062	0.051	0.011	0.06	0.96	123	
15	0.223	0.133	0.090	2.47	0.01	244	**

Table S1: Seed likelihood of resected and non-resected areas. Only ROIs leading to non-null spreading are included in the analysis.

	Diff.	t	p	Sig.
Best vs RA	0.061	1.45	0.17	
Best vs ⟨RA⟩	0.281	6.70	$< 10^{-4}$	*
Best vs RND	0.341	7.44	$< 10^{-5}$	*
RA vs ⟨RA⟩	0.220	5.47	$< 10^{-4}$	*
RA vs RND	0.280	4.62	$3 \cdot 10^{-4}$	*
⟨RA⟩ vs RND	0.060	1.70	0.11	

Table S2: Comparison of the goodness of fit with different seed definitions. Best stands for the best individual seed, RA for the resected area, ⟨RA⟩ for the average fit of the RA ROIs considered as individual seeds, and RND for random resections of the same size as the RA. To perform the comparison we used a paired t-test between the individual patient fits. $df = 14$ in all cases.

4 Virtual resection analysis

4.1 Seed regions: Recursive method

In order to perform the virtual resection analysis, we identified the most-likely seeds of sizes $1-5$ following a recursive method as illustrated in figure S4A, where for each seed size S we considered as possible seed regions the set of nodes formed by the optimal seed of size $S-1$ and one of the remaining nodes in the network. In S4B we illustrate the goodness-of-fit as a function of the seed size for each patient.

4.2 Statistical analyses

In this section we report on the details of the statistical analyses in section 2.5: “Virtual resection analysis” of the main text. Table S3 corresponds to the comparison of the effect of VRs of increasing size between the SF and NSF groups (corresponding to the analysis shown in figure 7 of the text). The data corresponding to this analyses are shown in figure S5, where each panel corresponds to a different seed size. Finally, table S4 indicates the statistical details of the step-wise linear regression analysis (table 1 and figure 8 of the main text).

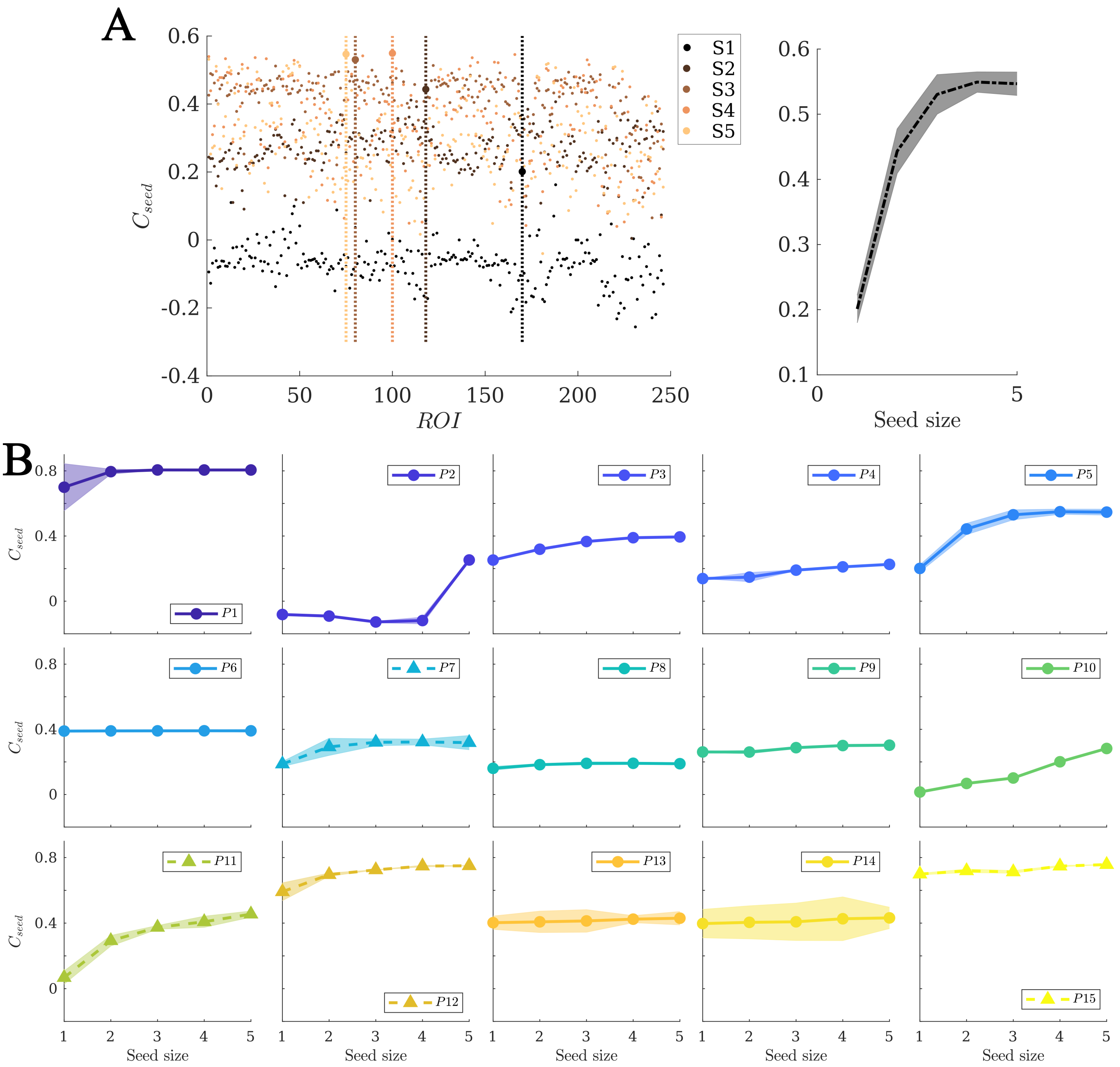

Figure S4: Recursive seeds for the virtual resection analysis. Panel **A** Illustrates the recursive procedure for one patient (case 5). Seeds of increasing sizes are derived following a recursive procedure (left panel). First, the best single seed is identified ($S1$). Then, seeds of size 2 formed by the best single seed and one of the remaining (in this case) 245 ROIs are considered ($S2$), and the pair leading to the best model fit (C_{seed}) is selected. This procedure is iterated until seeds of size 5 are obtained. The resulting goodness-of-fit as a function of the seed size S is illustrated in the right panel. The shaded bars show the uncertainties associated with the SIR dynamics. In the right panel the errorbars are not shown to aid visibility. The dotted vertical lines mark the best new ROI for each seed size. Panel **B** shows the $C_{\text{seed}}(S)$ curves for all patients.

S	SF	NSF	diff	t	p
1	−1.13	−1.87	0.740	1.61	0.13
2	−1.02	−1.44	0.414	1.48	0.16
3	−1.12	−1.57	0.448	1.62	0.12
4	−1.24	−1.55	0.314	1.34	0.2
5	−1.25	−1.45	0.200	0.81	0.4

Table S3: Comparison of the effect of virtual resections, as given by the normalized decrease in total spreading, $\delta_{\mathrm{VR}} = (IR_{\mathrm{BS}} - IR_{\mathrm{VR}})/\mathrm{IR}_{\mathrm{BS}}$, between the SF and NSF groups, for different seed sizes S. We report here (and in figure S5) on $\log(\delta_{\mathrm{VR}})$, since it displays better the different amount of spreading for different patients. $df = 13$ in all cases. diff indicates the difference between the groups, t the t-statistic and p the corresponding p-value. These results correspond to the analysis shown in figure 7 in the main text and in figure S5.

Adj. Variables	Estimate	SE	t	p
Intercept	−1.63	0.11	−15.4	$3 \cdot 10^{-24}$
S_{RA}	$4.64 \cdot 10^{-2}$	$1.1 \cdot 10^{-2}$	4.26	$6 \cdot 10^{-5}$
$\mathrm{BC}_{\mathrm{seed,VR}}$	$-4.26 \cdot 10^{-4}$	$1.3 \cdot 10^{-4}$	−3.33	$1 \cdot 10^{-3}$
$\Delta\mathrm{BC}_{\mathrm{seed}}$	$2.22 \cdot 10^{-3}$	$5.1 \cdot 10^{-4}$	4.33	$5 \cdot 10^{-5}$

Table S4: Results from the step-wise linear regression analysis. As independent variables we considered the 11 model and network parameters specific in Table 1 of the main text (spreading ratio, centrality metrics of the RA and seed). Only three variables survived: the size of the RA S_{RA}, the BC of the seed in the resected network, $\mathrm{BC}_{\mathrm{seed,VR}}$, and the change in BC of the seed due to the resection, $\Delta\mathrm{BC}_{\mathrm{seed}}$. The coefficients of the fit are indicated in this table, where SE stands for the standard error of the estimate, t is the t-statistic for a test that the coefficient is zero, and p is the corresponding p-value of the t-test. The main statistics of the fit are as reported in the main text (Table 1).

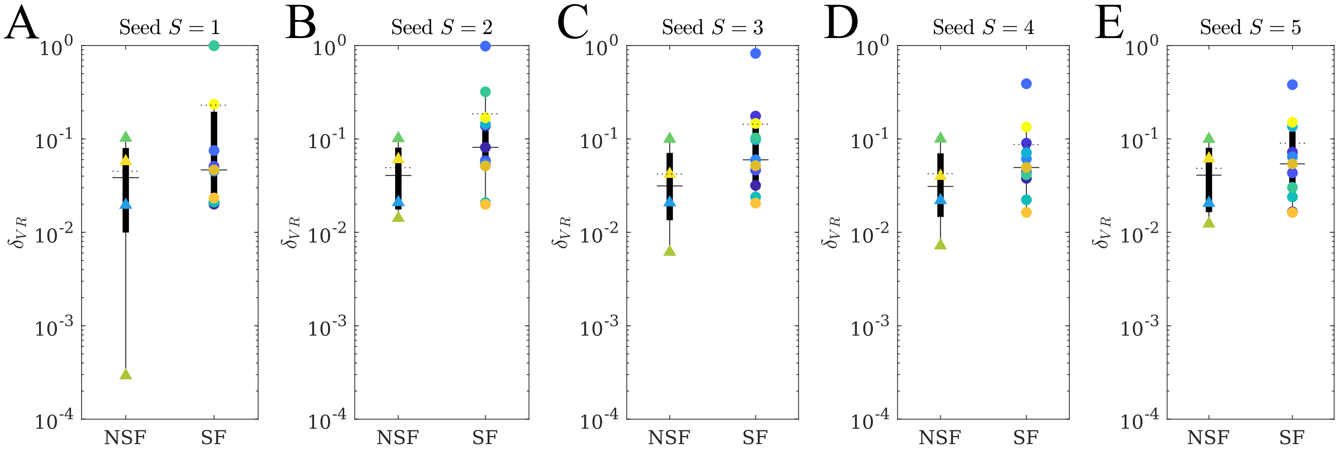

Figure S5: Effect of virtual resections: group effect. Comparison between the normalized decrease in spreading due to the VR of the RA, δ_{VR}, between the SF and NSF groups. Each panel corresponds to a different seed size as indicated in the header. Mean (median) values are given by dotted (solid) lines. Each data point corresponds to a different patient. The statistical details of the analyses are indicated in table $S3$.
